# Body position for preventing ventilator-associated pneumonia for critically ill patients: a systematic review and network meta-analysis

**DOI:** 10.1186/s40560-022-00600-z

**Published:** 2022-02-22

**Authors:** Diana P. Pozuelo-Carrascosa, Ana Isabel Cobo-Cuenca, Juan Manuel Carmona-Torres, Jose Alberto Laredo-Aguilera, Esmeralda Santacruz-Salas, Ruben Fernandez-Rodriguez

**Affiliations:** 1 Faculty of Physiotherapy and Nursing of Toledo, Department of Nursing, Physiotherapy and Occupational Therapy, University of Castilla-La Mancha, 45005 Toledo, Spain; 2 Multidisciplinary Research Group in Care (IMCU), University of Castilla-La Mancha, 45005 Toledo, Spain; 3 Health and Social Research Center (CESS), University of Castilla-La Mancha, Cuenca, Spain; 4 Maimónides Biomedical Research Instituto of Córdoba (IMIBIC), 14004 Córdoba, Spain

**Keywords:** Body position, Prone, Supine, Semi-recumbent, Ventilator-associated pneumonia

## Abstract

**Background:**

The evidence about the best body position to prevent ventilator-associated pneumonia (VAP) is unclear. The aim of this study was to know what the best body position is to prevent VAP, shorten the length of intensive care unit (ICU) and hospital stay, and reduce mortality among patients undergoing mechanical ventilation (MV).

**Methods:**

We performed a network meta-analysis of randomized controlled trials including intubated patients undergoing MV and admitted to an ICU. The assessed interventions were different body positions (i.e., lateral, prone, semi-recumbent) or alternative degrees of positioning in mechanically ventilated patients.

**Results:**

Semi-recumbent and prone positions showed a risk reduction of VAP incidence (RR: 0.38, 95% CI: 0.25–0.52) and mortality (RR: 0.70, 95% CI: 0.50–0.91), respectively, compared to the supine position. The ranking probabilities and the surface under the cumulative ranking displayed as the first best option of treatment the semi-recumbent position to reduce the incidence of VAP (71.4%), the hospital length of stay (68.9%), and the duration of MV (67.6%); and the prone position to decrease the mortality (89.3%) and to reduce the ICU length of stay (59.3%).

**Conclusions:**

Cautiously, semi-recumbent seems to be the best position to reduce VAP incidence, hospital length of stay and the duration of MV. Prone is the most effective position to reduce the risk of mortality and the ICU length of stay, but it showed no effect on the VAP incidence.

*Registration* PROSPERO CRD42021247547

**Supplementary Information:**

The online version contains supplementary material available at 10.1186/s40560-022-00600-z.

## Introduction

Ventilator-associated pneumonia (VAP) is a hospital-acquired pneumonia that develops in patients undergoing invasive mechanical ventilation (MV) for at least 48 h. [1] Although this disease is theoretically avoidable, VAP is one of the most common hospital-acquired infections in intensive care units (ICUs) [2], leading to increased mortality, ICU length of stay and healthcare costs [2–4].

The presence of an endotracheal tube is one of the main risk factors for the development of VAP because it interferes with the normal protective upper airway reflexes, irritates the respiratory mucosa, increases the amount of mucus, and promotes microaspiration of contaminated oropharyngeal secretions [5]. Some physical interventions have been shown to be effective for reducing the incidence of VAP, such as subglottic secretion drainage [6], continuous cuff pressure monitoring [7] and certain body positions [8], among others.

Positioning refers to the use of body position as a specific treatment technique, usually employed in combination with other physiotherapy techniques [9]. In the ICU, the patient’s body position may be intended to improve ventilation/perfusion, increasing the lung volumes or the clearance of airway secretions with the aid of gravity, among others [9]. For preventing VAP, a semi-recumbent position (i.e., elevation of the head of bed to 30–45°) has been extensively studied as a simple strategy for patients undergoing MV and is a recommendable measure in several clinical practice guidelines [8, 10–12]. This position can help reduce gastroesophageal reflux and avoid the entry of these gastric contents and contaminated oropharyngeal secretions into the lower airway, thus preventing VAP [13].

Although it seems that the semi-recumbent position is better in preventing VAP than the supine position [8], it has been suggested that other body positions, such as the prone position, could improve the outward drainage of biofluids and respiratory secretions, preventing the translocation of pathogens into the lower airway [14]. Moreover, the lateral position has been extensively considered in animals but not so much in humans, suggesting that the horizontal position of the endotracheal tube (external end below the tracheal level) and positioning the patient in the lateral–horizontal position, such as the recovery position, could be effective for reducing residual gastric volume [15] and avoiding lung infections [16, 17].

To date, several systematic reviews have separately synthesized the effects of different body positions, such as semi-recumbent, prone or lateral-Trendelenburg positions, to reduce the incidence of VAP [8, 14, 18–20]; nevertheless, evidence aimed at directly comparing the effectiveness of several body positions (i.e., lateral, prone, semi-recumbent and supine) to prevent VAP is still scarce. For this purpose, a network meta-analysis (NMA) is an ideal approach that allows us to compare the estimated pooled effect sizes (ES) from indirect comparisons of interventions that have not been compared in a head-to-head manner. Thus, it can comparatively estimate the effect of different body position interventions (i.e., lateral, prone, semi-recumbent and supine) on the VAP incidence, duration of MV, ICU/hospital length of stay and mortality among mechanically ventilated patients. Therefore, the research question for this NMA was which body position is the most effective for preventing VAP and for reducing mortality, the duration of MV and the ICU/hospital length of stay among intubated patients receiving MV.

## Methods

This NMA was registered at the International Prospective Register of Systematic Reviews—PROSPERO (CRD42021247547). In addition, this study was performed in accordance with the Preferred Reporting Items for Systematic Reviews and Meta-Analyses for Network-Meta-analyses (PRISMA–NMA) [21], and we also followed the recommendations of the Cochrane Handbook for Systematic Reviews of Interventions [22].

### Search strategy

We performed an electronic search using the following online databases from their inception to May 2021: Web of Science, EMBASE (via Scopus), Cochrane Database of Systematic Reviews, and MEDLINE (via PubMed). In addition, the reference lists of published full-text systematic reviews and/or meta-analyses were manually examined for relevant studies. The search was performed via the following medical subject headings (MeSH) and keywords and combined with Boolean operators: “body position”, “position”, “prone”, “Trendelenburg”, “supine”, “semi-recumbent”, “semirecumbent”, “prevention” “prevent*”, and “ventilator-associated pneumonia”. The search strategy was adapted for each database. No restrictions by publication year or country of study were made. This electronic search was conducted by DPP-C and RF-R, and any differences were resolved by discussion with a third reviewer (AIC-C).

### Study selection and data extraction

After removing duplicate retrieved records, two reviewers (DPP-C and RF-R) independently screened the titles and abstracts. Then, the reviewers evaluated the full-text articles, and when any discrepancy between the two independent reviewers occurred, a third coauthor was consulted to resolve it (AIC-C).

We included randomized controlled trials (RCTs) that met the following inclusion criteria: patients undergoing endotracheal intubation and mechanical ventilation for at least 48 h and reported data on VAP incidence.

We included RCTs comparing different body positions or alternative degrees of positioning of mechanically ventilated patients: supine, semi-recumbent, prone or lateral. The main study outcome measure was the incidence of VAP (clinically suspected or microbiologically confirmed), and the secondary outcome variables were ICU length of stay, hospital length of stay, duration of MV and mortality.

Trials with quasi-experimental, cluster randomization and crossover designs and only published as abstracts were excluded. In addition, unpublished studies or those including repeated data were excluded. No language restrictions were applied.

Data from the included RCTs were extracted through a standard data extraction form, including (1) first author; (2) year of publication; (3) country; (4) characteristics of the participants; (5) outcomes: incidence of VAP (clinically suspected or microbiologically confirmed), ICU length of stay, hospital length of stay, duration of MV and mortality; (6) characteristics of the treatments: body position (supine, semi-recumbent, prone or lateral), angles and hours per day in this position; and (7) other related cointerventions.

### Quality assessment

#### Methodological quality

Two independent researchers assessed the risk of bias of the included studies, and a third reviewer was consulted to resolve discrepancies. For this, we used the Cochrane Collaboration Risk of Bias Tool 2 (RoB2) [23] to assess the following items of each included study: (1) the randomization process, (2) deviations from the intended interventions, (3) the presence of missing outcome data, (4) measurement of the outcome and (5) selection of the reported results. In addition, overall bias was rated as “low risk”, “some concerns” or “high” risk of bias.

#### Quality of evidence

We used the Grading of Recommendations, Assessment, Development, and Evaluation (GRADE) tool [24] to assess the quality of the available evidence. Each of the included outcomes could be scored as high, moderate, low or very low evidence value, depending on the design of the studies, risk of bias, inconsistency, indirect evidence, imprecision and publication bias. These factors could increase or decrease the quality of the evidence: (1) risk of bias (downgraded once when < 75% of the analysed studies were at low risk of bias); (2) inconsistency (downgraded once when the *I*^2^ > 50%); (3) indirect evidence (such as indirect population, intervention, control or outcomes); (4) imprecision displayed in wide confidence intervals; and (5) the presence of publication bias also downgraded the quality of the evidence [24–26].

To produce the “summary of findings” tables for each main pairwise comparison, we used GRADEpro-GDT software (www.gradepro.org).

### Data synthesis and analysis

#### Categorization of available evidence

Body position interventions were determined as follows:

- The supine position was defined as a body position with a head-of-bed elevation angle of 0–10° [8]. In addition, when the study reported a supine position, but the angle was greater than 10°, but less than 30°, this position was also categorized in the supine group.
- The semi-recumbent position was defined as upright positioning of the head and torso at an angle ≥ 30° [8, 27]. Different angles ≥ 30° were considered in this category.
- The prone position was defined as the posture of an individual lying face down, regardless of the length of time the position was maintained.
- The lateral–Trendelenburg position was defined as a position in which the patient was positioned in a semilateral position, such as the recovery position, with the head of the bed tilted 5–10° in the Trendelenburg position [28].

#### Statistical analysis

We performed the NMA according to the PRISMA–NMA statement [21], distinguishing the following phases. First, we presented the strength of the available evidence using a network diagram for direct comparisons between the different interventions for each outcome [29]. Random-effects pairwise meta-analyses were performed for VAP incidence, mortality, duration of MV, or length of ICU/hospital stay comparing the different treatment options. For VAP incidence and mortality, the risk ratio (RR) with 95% confidence intervals (CI) was calculated, while for the duration of MV and ICU/hospital length of stay, the mean differences (MD) with 95% CI were calculated between groups (intervention versus control-supine position) and pooled using the random-effects DerSimonian–Laird method [30].

Second, to perform the NMA, we conducted simultaneous comparisons of several interventions, creating a connected network using the totality of the available evidence (direct and indirect comparisons) [29, 31]. For each outcome, we reported the mean treatment effect with its 95% CI (standardized mean differences for VAP and mortality and raw MD for the duration of MV and the ICU/hospital length of stay) of all interventions relative to the other interventions, including the control and the estimated common network-specific heterogeneity parameter [32]. The *I*^2^ statistic was used to examine the statistical heterogeneity according to the following values: not important (0–40%), moderate (30–60%), substantial (50–90%) and considerable (75–100%) [33]. Furthermore, the τ^2^ statistic was calculated using the following values for its interpretation: 0.04 low, 0.14 moderate and 0.40 as a substantial degree of clinical relevance of heterogeneity [33]. In addition, the relative ranking of the different body positions was calculated for each outcome using the distribution of the ranking probabilities and the surface under the cumulative ranking (SUCRA); in this sense, the best intervention would obtain a value close to 1, and the worst would obtain a value close to 0. Following the recommendations of Brignardello-Petersen et al. [34] for NMA scenarios in which most evidence is indirect, the probability of each intervention (i.e., supine, prone, semi-recumbent or lateral) being the most effective was depicted using rankograms. The consistency was evaluated by checking by checking that intervention effects estimated from direct comparisons were consistent with those estimated from indirect comparisons. Confidence was assessed with the Confidence In Network Meta-Analysis (CINeMA) web tool [35]; for this, relative effect estimates below − 0.20 and above 0.20 were considered clinically important for incidence of VAP and mortality outcomes, and relative effect estimates higher than 2 days for hospital length of stay and 1 day for ICU length of stay and duration of MV. For the transitivity assessment, we checked that all participants in the studies included in the NMA had similar baseline important clinical and methodological characteristics (age, gender, Acute Physiology and Chronic Health Disease Classification System II [APACHE II] or Glasgow Coma Scale [GCS], Simplified Acute Physiology Score [SAPS] and PaO_2_/FiO_2_) that might modify the treatment effect [36, 37]. In addition, the small study effect was analysed, and a network funnel plot was used to visually inspect the criterion of symmetry [38].

The same process as mentioned above was employed in a subgroup analysis to assess the best angle degrees of semi-recumbency to prevent VAP in patients undergoing MV and admitted to the ICU.

All analyses were conducted with Stata V.15.0 (Stata), and with the CINeMA software. [35].

## Results

**Fig. 1:**
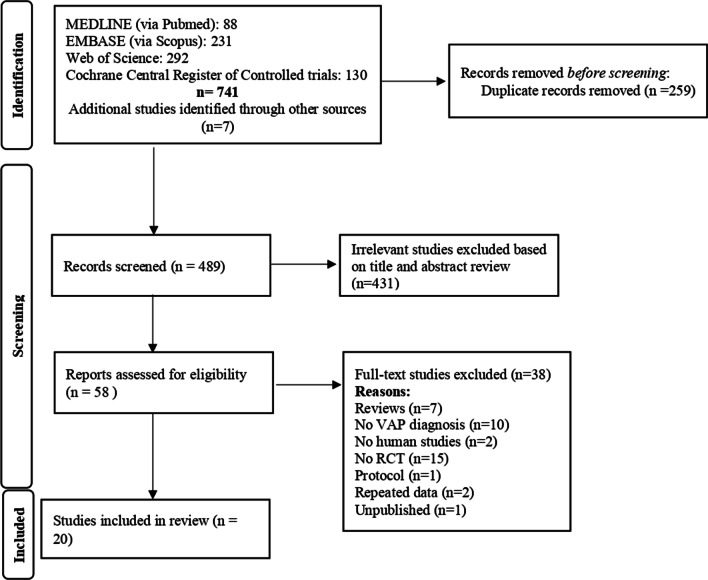
Literature search: Preferred Reporting Items for Systematic Reviews and Meta-Analyses (PRISMA) consort diagram

**Fig. 2:**
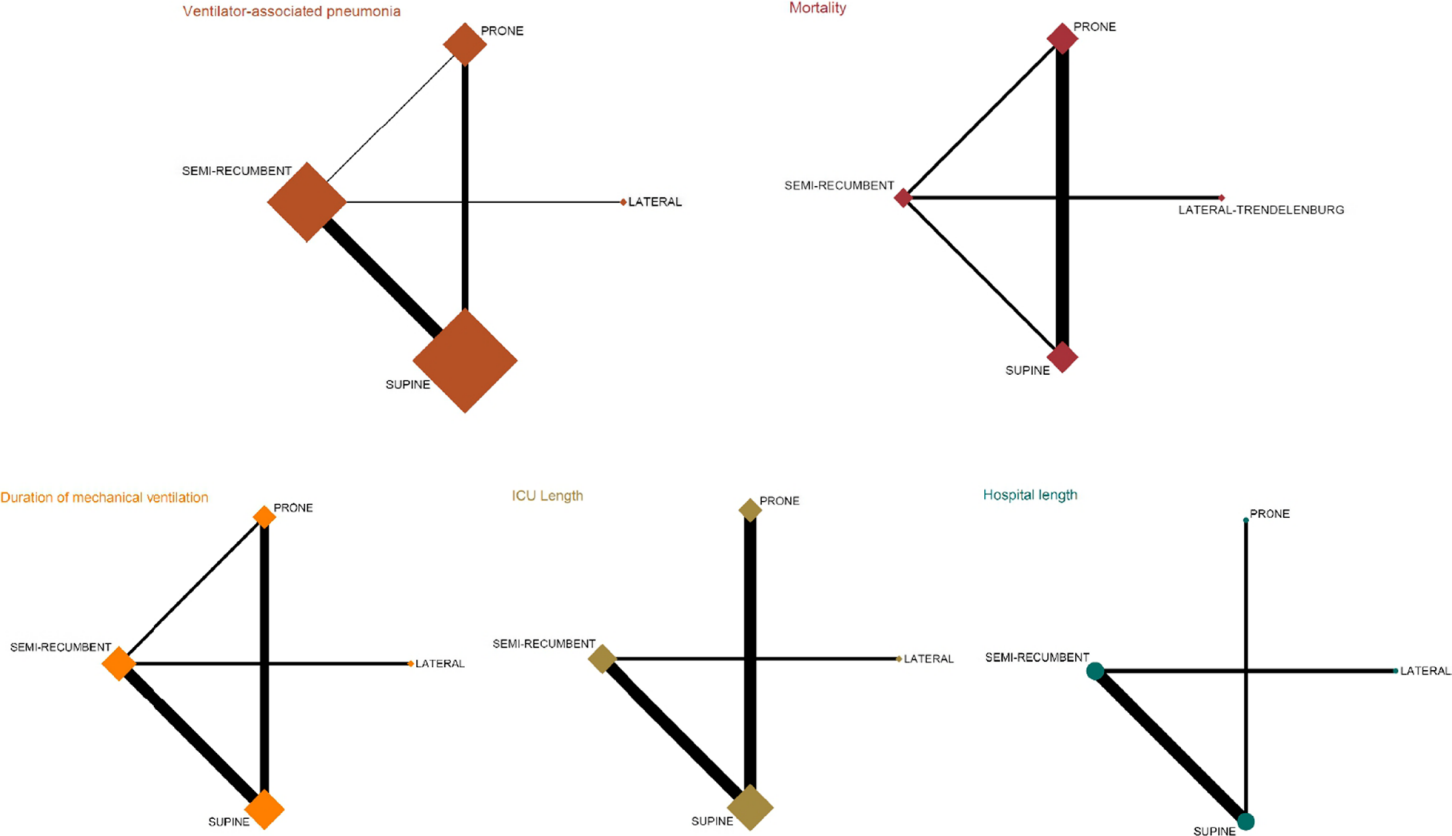
Network geometry graphs for changes on ventilator-associated pneumonia, mortality, ICU/hospital length of stay and duration of mechanical ventilation

The electronic search retrieved 741 results. After excluding duplicates and irrelevant studies based on the title and abstract, a total of 58 studies were selected for the full-text assessment. We manually inspected the reference lists of the systematic reviews and/or meta-analyses obtained in the electronic search to identify additional studies. Finally, 20 RCTs were included (Fig. 1); among them, six studies analysed the prone versus supine comparison [39–44], 11 studies analysed the semi-recumbent versus supine comparison [45–55], one study assessed the effect of the semi-recumbent position versus the lateral–Trendelenburg position [28], one study compared the effect of semi-recumbent versus prone positions [56], and finally, one study compared the effectiveness of different angle degrees of the semi-recumbent position to prevent VAP [57]. According to the outcome assessment, 20 studies analysed the effect of positioning on VAP, 10 studies on mortality, 9 studies assessed the effect of positioning to reduce the duration of MV and the ICU length of stay, and finally, 5 studies reported data about the hospital length of stay (Table 1 and Fig. 2). The results of everyone included study are available in Additional file 1.

**Table 1:** Characteristics of studies included

Study, year	Country	Population	Interventions	Diagnosis of VAP	Care Bundle
Ayzac et al. 2016 [39]	France	Adults, invasive MV for ARDS, with severity criteria PaO_2_/FiO_2_ < 150 mmHg under FiO_2_ ≥ 0.6, PEEP ≥ 5 cm H_2_O, and VT = 6 ml/kg predicted body weight in previous 36 h, fulfilled after a 12–24 h stabilization period	ARM 1: Supine position; *n* = 229 ARM 2: Prone position, for at least 16 h consecutive; *n* = 237	BAL ≥ 10^4^ cfu/ml Tracheal aspirate ≥ 10^7^ cfu/ml Wimberly brush ≥ 10^3^ cfu/ml	Lung protective MV: VT = 6 ml/kg predicted body weight, neuromuscular blockade, and sedation, and weaning from MV
Bassi et al. 2017	Spain, Italy, Germany, Croatia, USA	Patients ≥ 18 years, expected to be on MV for at least 48 h, within 12 h from endotracheal intubation	ARM 1: Lateral Trendelenburg position; *n* = 194 ARM 2: Semi-recumbent position 30° angle; *n* = 201	BAL or mini-BAL cultures ≥ 10^4^ cfu/ml PSB ≥ 10^3^ cfu/ml	Active humidification of respiratory gases. Every 6 h, patients were rotated from one side to the other
Beuret et al. 2002 [40]	France	Invasive oral MV for coma, Glasgow ≤ 9. Initial settings were selected to obtain a VT = 10 ml/kg. They were further adjusted to PaCO_2_ = 5–40 mmHg, PEEP = 5 cm H_2_O	ARM 1: Supine position, head and trunk positioned at 0–20° angle; *n* = 28 ARM 2: Prone position, strictly horizontal, 4 h/day; *n* = 25	PSB > 10^3^ cfu/ml	NR
Cai et al. 2006	China	Adults admitted in ICU	ARM 1: Supine position, 0° angle; *n* = 27 ARM 2: Semi-recumbent position, 30° angle; *n* = 27	Clinically suspected pneumonia: new, persistent or progressive radiographic infiltrate with at least two criteria: fever (Tª > 38 ℃ or < 35 ℃); leukocytosis or leucopenia (leucocytes > 10 × 10^9^/L or < 3 × 10^9^/L); and positive culture of tracheal secretion	NR
Drakulovic et al. 1999 [46]	Spain	Patients intubated and with MV in two ICUs: respiratory and medical	ARM 1: Supine position, 0° angle; *n* = 47 ARM 2: Semi-recumbent position, 45° angle; *n* = 39	Tracheobronchial aspirate > 10^5^ cfu/ml BAL > 10^4^ cfu/ml PSB > 10^3^ cfu/ml	Sterile endotracheal suctioning; no change of MV tubing systems; stress ulcer prophylaxis; antacid medication
Fernández et al. 2008 [42]	Spain	Intubated adult patients within 48 h of ARDS diagnosis	ARM 1: Supine position; *n* = 19 ARM 2: Prone position; at least 20 h/day; *n* = 21	NR	Ventilator pattern, sedation and weaning protocoled
Guérin et al. 2004	France	Patients > 18 years, with MV through either oral or nasal tracheal intubation or tracheostomy; a PaO_2_/FIO_2_ of 300 or less. Expected duration of MV longer than 48 h	ARM 1: Semi-recumbent position; 30° angle *n* = 378 ARM 2: Prone position at least 8 h/day; *n* = 413	BAL ≥ 10^4^ cfu/ml Wimberley brush ≥ 10^3^ cfu/ml	Periodic left and right lateral decubitus
Hadi Hassankhan et al. 2017 [57]	Iran	Patients intubated with MV for ≥ 7 days (MODE = SIMV, VT = 6–8 ml/kg, PEEP = 2.5–7.5 cm H_2_O, FiO_2_ = < 50%, RR = 6–12/min)	ARM 1: Semi-recumbent position 45° angle; *n* = 11 ARM 2: Semi-recumbent position 60° angle; *n* = 10	Sputum culture obtained by endotracheal suction technique	Oral and endotracheal suctioning routine; endotracheal cuff monitored; endotracheal with dorsal lumen and continuous suction; position changes every 2 h; prevention of stress ulcer; heparin and mouth washed with chlorhexidine
Hang et al. 2012 [47]	China	Adult critical ventilated patients in ICU	ARM 1: Supine position, 0° angle; *n* = 19 ARM 2: Semi-recumbent position, 30° to 45° angle; *n* = 20	Clinically suspected pneumonia: new, persistent or progressive radiographic infiltrate with at least two criteria: fever (Tª > 38 ℃ or < 35 ℃); leukocytosis or leucopenia (leucocytes > 10 × 10^9^/L or < 3 × 10^9^/ L); and positive culture of tracheal secretion	Enteral feeding and sucralfate or H2 antagonists for stress ulcer prophylaxis
Hu et al. 2012 [48]	China	Adult critical ventilated patients in ICU	ARM 1: Supine position, 0° angle; *n* = 43 ARM 2: Semi-recumbent position, 30° to 45° angle; *n* = 43	Clinically suspected pneumonia, but not definition	Enteral feeding, H2 antagonists for stress ulcer prophylaxis, and use of antibiotic prophylaxis
Keely et al. 2007	UK	Adult critical ventilated patients in ICU	ARM 1: Supine position, 25° angle; *n* = 13 ARM 2: Semi-recumbent position, 45° angle; *n* = 17	BAL Tracheobronchial aspirate PSB	Standard ICU practices: nasogastric tubes for enteral feeding and parenteral nutrition; gastric ulcer prophylaxis; no change of ventilator tubing
Loan et al. 2012	Vietnam	Adults and children (aged ≥ 1 year) admitted to the ICU with a clinical diagnosis of severe tetanus	ARM 1: Supine position, 0° angle; *n* = 106 ARM 2: Semi-recumbent position, 30° angle; *n* = 104	No-BAL ≥ 10^5^ cfu/ml	Tetanus antitoxin, benzodiazepines to control muscle spam and hypertonia
Mancebo et al. 2006 [42]	Spain and Mexico	Patients > 18 years, intubated with MV, and severe ARDS diagnosis	ARM 1: Supine position; *n* = 60 ARM 2: Prone position; at least 20 h/day; *n* = 76	NR	NR
Tahereh Najafi Ghezeljeh et al. 2017 [51]	Iran	Age > 18 years, no history of VAP, hospitalized in the ICU, under MV support for 8 h after hospitalization, no injuries in the spine, and no pelvic unstable fracture	ARM 1: Supine position; *n* = 40 ARM 2: Semi-recumbent position 30° angle; *n* = 40 ARM 3: Semi-recumbent position 45° angle; *n* = 40	Mini BAL	Changing the position every 2 h, assessment of pressure areas, changing wet sheets, rinsing with chlorhexidine, tracheal suction
Van Nieuwenhoven et al. 2006 [52]	Netherlands	Adult patients intubated within 24 h of ICU admission and had an expected duration of VM of at least 48 h	ARM 1: Supine position, 10° angle; *n* = 109 ARM 2: Semi-recumbent position, 45° angle; *n* = 112	BAL ≥ 10^4^ cfu/ml	Sucralfate or H2 antagonists for stress ulcer prophylaxis. Enteral feeding via nasogastric tube
Voggenreiter et al. 2005 [43]	Germany	Multiple trauma patients (18–80 years; ISS ≥ 16) who were receiving MV with a PaO_2_/FiO_2_ ≤ 200 or with a PEEP ≥ 5 cm of water, and (if measured) a pulmonary capillary wedge pressure ≥ 18 mm Hg, or the absence of clinical evidence of left atrial hypertension and pulmonary infiltrates on chest x-ray	ARM 1: Supine position; *n* = 19 ARM 2: Prone position; at least 8 h/day and maximum 23 h/day; *n* = 21	BAL	NR
Watanabe et al. 2002 [44]	Japan	Patients admitted in ICU who underwent three-field lymphadenectomy, with PaO_2_/FIO_2_ < 200, PEEP > 5 cm H_2_O, on the fifth postoperative day	ARM 1: Supine position; *n* = 8 ARM 2: Prone position; *n* = 8	NR	NR
Wu et al. 2009 [53]	China	Adult critical ventilated patients in ICU	ARM 1: Supine position, 0° angle; *n* = 56 ARM 2: Semi-recumbent position, 30° to 60° angle; *n* = 56	Tracheobronchial aspirate > 10^5^ cfu/ml BAL > 10^4^ cfu/ml PSB > 10^3^ cfu/ml	Enteral feeding and use of antibiotic prophylaxis
Xue et al. 2012 [54]	China	Adult critical patients in ICU with VM > 48 h	ARM 1: Supine position, 0° angle; *n* = 48 ARM 2: Semi-recumbent position, 30° to 45° angle; *n* = 48	Clinically suspected pneumonia: new, persistent or progressive radiographic infiltrate with at least two criteria: fever (Tª > 38 °C or < 35 °C); leukocytosis or leucopenia (leucocytes > 10 × 10^9^/L or < 3 × 10^9^/L); and positive culture of tracheal secretion	NR
Yu et al. 2012 [55]	China	Adult critical ventilated patients in ICU	ARM 1: Supine position, 0° angle; *n* = 32 ARM 2: Semi-recumbent position, 30° angle; *n* = 33	Clinically suspected pneumonia: new, persistent or progressive radiographic infiltrate with at least two criteria: fever (Tª > 38 °C or < 35 °C); leukocytosis or leucopenia (leucocytes > 10 × 10^9^/L or < 3 × 10^9^/L); and positive culture of tracheal secretion	NR

*CFU* colony forming units, *VT* volume tidal, *MV* mechanical ventilator, *PEEP* positive end-expiratory pressure, *FiO*_*2*_ fraction of inspired oxygen, *BAL* bronchoalveolar lavage, *PSB* protected-specimen brush, *ISS* injury severity score, *PaO*_*2*_ partial pressure of arterial oxygen, *ARDS* acute respiratory distress syndrome, *NR* no reported, *ICU* intensive care unit

### Incidence of VAP

The direct comparisons showed a protective effect of the semi-recumbent versus supine position to prevent VAP (RR: 0.38, 95% CI: 0.25–0.52; *n* = 11). Although the prone position showed a beneficial effect relative to the semi-recumbent and supine positions, the effect estimates did not reach statistical significance (Table 2) (Additional file 2).

**Table 2:** Pooled effect sizes and 95% confidence interval (CI)

	Supine	Semi-recumbent	Lateral	Prone
Ventilator-associated pneumonia				
Supine		**0.38 (0.25 to 0.52)**	NA	0.79 (0.57 to 1.02)
Semi-recumbent	− 0.15 (− 0.30 to 0.01)		0.13 (0.02 to 1.03)	0.86 (0.66 to 1.11)
Lateral	− 0.18 (− 0.71 to 0.35)	− 0.04 (− 0.54 to 0.47)		NA
Prone	− 0.07 (− 0.27 to 0.14)	0.08 (− 0.16 to 0.32)	0.11 (− 0.45 to 0.67)	
Mortality				
Supine		0.83 (0.53 to 1.13)	NA	**0.71 (0.50 to 0.91)**
Semi-recumbent	− 0.05 (− 0.13 to 0.04)		1.27 (0.92 to 1.76)	1.03 (0.84 to 1.26)
Lateral	0.02 (− 0.15 to 0.18)	0.06 (− 0.08 to 0.21)		NA
Prone	− 0.09 (− 0.20 to 0.13)	− 0.05 (− 0.15 to 0.05)	− 0.11 (− 0.29 to 0.07)	
ICU length of stay				
Supine		1.02 (− 5.50 to 7.54)	NA	− 0.89 (− 6.49 to 4.72)
Semi-recumbent	1.09 (− 5.12 to 7.29)		− **1.25 (**− **1.60 to **− **0.90)**	NA
Lateral	− 0.16 (− 13.40 to 13.03)	− 1.25 (− 12.89 to 10.39)		NA
Prone	− 0.81 (− 7.72 to 6.11)	− 1.89 (− 11.19 to 7.39)	− 0.64 (− 15.33 to 14.24)	
Hospital length of stay				
Supine		− 6.94 (− 20.30 to 6.43)	NA	5.80 (− 8.25 to 19.85)
Semi-recumbent	− 7.29 (− 22.74 to 8.17)		− **1.25 (**− **1.92 to **− **0.58)**	NA
Lateral	− 8.54 (− 39.14 to 22.07)	− 1.25 (− 27.67 to 25.17)		NA
Prone	5.79 (− 24.41 to 36.01)	13.09 (− 20.84 to 47.02)	14.33 (− 28.67 to 57.34)	
Duration of mechanical ventilation				
Supine		− 3.36 (− 7.81 to 1.09)	NA	− 2.83 (− 8.03 to 2.36)
Semi-recumbent	− **3.26 (**− **6.31 to **− **0.20)**		**0.50 (0.27 to 0.73)**	− 0.40 (− 1.54 to 0.74)
Lateral	− 2.76 (− 9.43 to 3.91)	0.50 (− 5.42 to 6.43)		NA
Prone	− 3.28 (− 8.05 to 1.49)	− 0.03 (− 4.70 to 4.65)	− 0.52 (− 8.08 to 7.03)	

Upper right triangle gives the pooled risk ratios (for ventilator-associated pneumonia and mortality) and mean differences (for ICU/hospital length of stay and duration of mechanical ventilation) from pairwise comparisons (column intervention relative to row), lower left triangle pooled standardized mean differences (for ventilator-associated pneumonia and mortality) and raw mean difference (for ICU/hospital length of stay and duration of mechanical ventilation) from the network meta-analysis (row intervention relative to column). Bold values denote statistical significance at p<0.05.

*NA* not available, *ICU* intensive care unit

Indirect comparisons showed a positive trend towards a decrease in the incidence of VAP in all body positions when they were compared with the supine position; nevertheless, none of these results reached statistical significance (Table 2).

### Mortality

Direct comparisons revealed that the prone position had a positive effect on the reduction of mortality compared to the supine position (RR: 0.71, 95% CI: 0.50–0.91; *n* = 4). The semi-recumbent position showed a lower risk of mortality than the supine, lateral and prone positions, but these effect estimates did not reach statistical significance (Table 2) (Additional file 3). Indirect comparisons revealed that the worst position to reduce mortality was the lateral–Trendelenburg relative to the semi-recumbent, prone, and supine positions; nevertheless, none of these ESs of these comparisons reached statistical significance (Table 2).

### ICU length of stay

The results of the meta-analysis revealed that patients positioned in the lateral Trendelenburg position spent less time (1.25 days) in the ICU than patients positioned in the semi-recumbent position (MD: − 1.25, 95% CI: − 1.60 to − 0.90; *n* = 1). The NMA results revealed no significant reductions in the ICU length of stay for any position (Table 2).

### Hospital length of stay

As previously shown in the ICU length of stay results, the lateral–Trendelenburg position achieved a reduction in the hospital length of stay compared to the semi-recumbent position (MD: − 1.25, 95% CI: − 1.92 to − 0.58; *n* = 1). Similarly, in the NMA analyses, the hospital length of stay was not reduced by any specific position (Table 2).

### Duration of mechanical ventilation

The duration of MV was higher in patients positioned in the lateral Trendelenburg position than in those positioned in the semi-recumbent position (MD: 0.50, 95% CI: 0.27 to 0.73; *n* = 1). Nevertheless, the NMA results showed a lower duration of MV in patients positioning in the semi-recumbent position than in those in the supine position (raw MD: − 3.26, 95% CI: − 6.31 to − 0.20; 9 comparisons) (Table 2).

### Treatment ranking

**Fig. 3:**
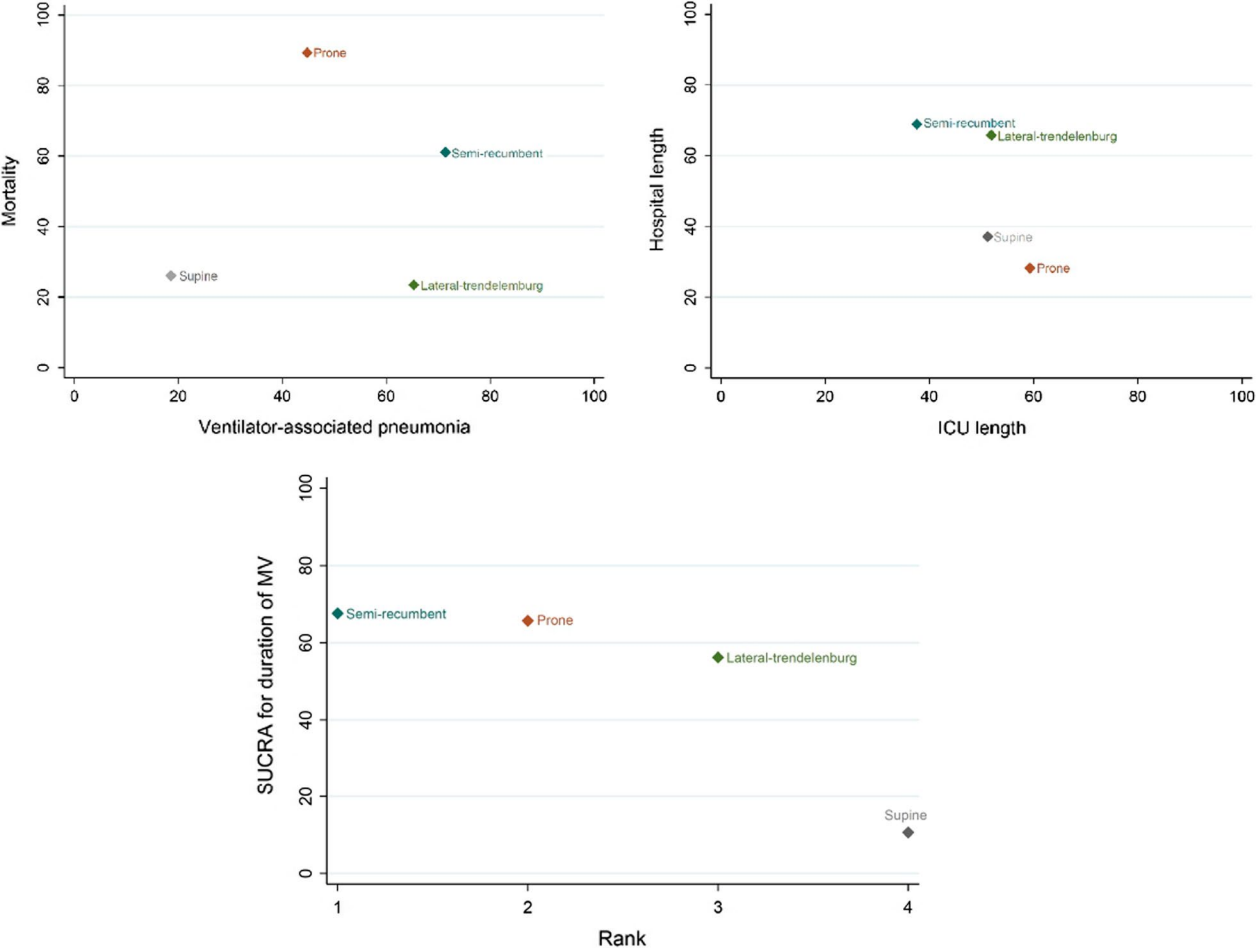
Treatment ranking for each assessed outcome (incidence of ventilator-associated pneumonia, mortality, hospital, and ICU length of stay and duration of mechanical ventilation)

The first- and second-best options according to their SUCRA values for the studied outcomes were the following treatment strategies: to reduce the incidence of VAP, the semi-recumbent position (71.4%) and lateral–Trendelenburg (65.3%); to decrease the mortality, the prone position (89.3%) and semi-recumbent (61.1%); to reduce the ICU length of stay, the prone position (59.3%) and lateral–Trendelenburg (51.9%); to reduce the hospital length of stay, the semi-recumbent position (68.9%) and lateral–Trendelenburg (65.8%); and to reduce the duration of MV, the semi-recumbent (67.6%) and prone positions (65.7%) (Fig. 3) (Additional file 4).

### Heterogeneity, sensitivity, and small study effect analyses

The sensitivity analysis did not show significant changes when the individual study data were removed from any comparison analysis.

The heterogeneity for pairwise comparisons was not important for the comparisons of prone and semi-recumbent positions versus supine position for VAP (*I*^2^ = 5.6% and *I*^2^ = 37.0%, respectively) and mortality outcomes (*I*^2^ = 0.0% and *I*^2^ = 15.2%). The comparison of semi-recumbent versus supine positions showed considerable heterogeneity for the duration of MV (*I*^2^ = 92.9%) and ICU (*I*^2^ = 91%) and hospital (*I*^2^ = 96.2%) length of stay outcomes (Additional file 5). The pairwise comparison between prone and supine positions showed no important heterogeneity for the duration of MV (*I*^2^ = 0.0%) and substantial heterogeneity for the ICU length of stay outcomes (*I*^2^ = 66.4%).

Funnel plot asymmetry and Egger’s test did not show a small-study effect on any outcome: VAP (*p* = 0.089), mortality (*p* = 0.055), ICU length of stay (*p* = 0.701), hospital length of stay (*p* = 0.428), and duration of MV (*p* = 0.227) (Additional file 6).

### Transitivity and consistency assessment

The population included in the control groups of the different interventions was similar in the baseline distribution of the potential effect modifiers analysed (no significant differences in age, gender, number of events or sample size). Nevertheless, some potential modifiers, such as the Acute Physiology and Chronic Health Disease Classification System II (APACHE II) or Glasgow Coma Scale (GCS), could not be analysed due to the scarcity of studies reporting these variables (Additional file 7). The results of inconsistency and coherence are displayed in Additional file 8.

### Risk of bias

The overall risk of bias was “some concerns” for most of the included articles (75%). When the studies were analysed by individual domains, 55% of them had “low risk of bias” in the randomization process; nevertheless, 60% of studies had “some concerns” in the domain assessing deviations from the intended interventions; the presence of missing outcomes data domain had a “low risk of bias” in 70% of the studies. Fifty percent of the studies analysed obtained a “low risk of bias”, and 40% had a “high risk of bias” in the measurement of the outcome domain. Finally, 95% of the studies had shortcomings in the selection of the reported results domain (Additional file 9).

### Quality assessment

Quality assessment and grade of recommendation were evaluated with GRADE-pro tool, and there was low certainty of the evidence for the semi-recumbent versus supine position for the VAP outcome; for the rest of body positions assessed for all outcomes the grade evidence profile demonstrated very low confidence in all estimates of effects (Additional files 10, 11).

### Subgroup analysis

A subgroup analysis by different grades of semi-recumbent position was performed to establish whether the angle grades could influence the VAP incidence. Thus, the meta-analysis results showed that any angle greater than or equal to 30° of head elevation was effective in reducing the incidence of VAP when compared to the supine position. The results of the NMA showed a significant reduction in the VAP incidence in the 30–45° head-of-bed elevation group compared with the 30–60° angle of the semi-recumbent position (SMD: − 0.66, 95% CI: − 1.29 to − 0.03; *n* = 14) (Additional file 12). The higher SUCRA was for the 60° angle of head-of-bed elevation, followed by the 30–60° angle position (Additional file 13). The sensitivity analysis did not show any change in the overall SMD when the studies were removed one-by-one from the main analysis. A small-study effect was detected, showing a slightly asymmetrical funnel plot and Egger’s test *p* value = 0.003.

## Discussion

Our main findings were that the semi-recumbent position is effective for significantly reducing the incidence of VAP (62% RR reduction); in addition, the prone position seems to be the best position to reduce mortality in mechanically ventilated patients in the ICU, reducing the RR of mortality by 30% compared to the supine position.

Similar to our results, a previous Cochrane review concluded that the semi-recumbent position (30–60°) was an effective therapeutic tool to reduce the incidence of clinically suspected VAP (64% RR reduction); nevertheless, the reduction in microbiologically confirmed VAP did not reach statistical significance, probably because only three studies reported these data [8].

The semi-recumbent position has been classically used as the standard of care to avoid gastroesophageal reflux and prevent pulmonary aspiration and VAP [14]; nevertheless, this positioning measure has also been questioned. In this position, the contaminated secretions located on the cuff could pass into the lungs because of gravity, promoting the translocation of oropharyngeal pathogens into the lower respiratory tract [14, 58]. Despite this, a semi-recumbent position is recommended by several guidelines [11, 12] as a VAP preventive measure, and our results are consistent with them.

Although there is enough evidence supporting the semi-recumbent position to prevent VAP, the optimal degree of head-of-bed elevation remain unclear. In this sense, our subgroup analyses showed that the semi-recumbent position at 60° (followed by semi-recumbent at 30–60°) seems to be the best treatment option to reduce the incidence of VAP; nevertheless, these results should be interpreted cautiously because only five out of eleven included studies monitored and corrected the planned head-of-bed elevation angles [46, 50–53], and some of them failed in the adherence or registration of the recommended semi-recumbent angle position [50, 52]. In addition, although our results have shown the protective effect of higher angles of the semi-recumbent position, some authors have suggested that high degrees of head-of-bed elevation could increase the risk of sacral pressure sores [59] and haemodynamic instability [60], among others.

Prone positioning obtained a higher ranking probability of reducing the mortality in mechanically ventilated ICU patients, with a 30% RR reduction, but did not reduce the VAP incidence. A previous meta-analysis found contrary results, since the prone position achieved a reduction in the incidence of VAP but did not improve survival [19]. These discrepancies could be due to the higher number of studies included in our work, probably because 6 out of 20 studies [39, 41–44, 56] included were aimed for improvement oxygenation in acute respiratory disease syndrome (ARDS) patients, and not for preventing VAP. The prone position has been widely used in patients with ARDS to improve arterial oxygenation and to maintain a better ventilation/perfusion ratio [61]. Nevertheless, the results regarding the effect of the prone position on mortality are still controversial [19, 20, 62, 63], showing an increased risk of pressure ulcers and endotracheal tube obstruction or dislodging when compared to the supine position [19, 20, 64]. Even so, this position is recommended by several guidelines in patients with severe ARDS for more than 12 h per day [65, 66], although not as a VAP preventive measure [7, 14].

The lateral–Trendelenburg position reduced the ICU and hospital length of stay compared to the semi-recumbent position. Nevertheless, these results are based on only one study, which stopped the study after the second interim analysis due to the low incidence of VAP in the control group and the occurrence of adverse events in the lateral–Trendelenburg group [28]. This study was based on the hypothesis that the lateral–Trendelenburg position allows the tracheal and pulmonary axes to be oriented below horizontal, promoting mucus clearance and avoiding pulmonary aspiration [14]. In fact, a previous nonrandomized trial showed a trend to reduce the incidence of VAP in the lateral–horizontal position group compared to the semi-recumbent position group, and no serious adverse events occurred in patients positioned in lateral–horizontal decubitus; nevertheless, this study was not conclusive, which may be due to its small sample size. [67].

The NMA results displayed a significant reduction in the duration of MV in the semi-recumbent position group compared to the supine position, and the same trend was observed in the lateral and prone positions compared to the supine position, although without statistical significance.

The SUCRA results showed that the prone position was the most effective body positioning therapy to reduce mortality and ICU length of stay, while the semi-recumbent position was the most effective to reduce the duration of MV, hospital length of stay and VAP incidence.

The results of this study are consistent with the actual recommendations about positioning: (1) the semi-recumbent position is still widely recommended as a measure to prevent VAP; and (2) the prone position is mainly recommended in patients with severe ARDS, aimed at improving oxygenation and favouring mucus drainage.

Nevertheless the results should be interpreted cautiously, because one of the main limitations of this study, in addition to the inherent limitations of meta-analysis, is the not possibility of assessment of transitivity assumption, because only five included studies reported information of major effect modifiers, such as APACHE II, GCS or PaO_2_/FiO_2_, which could inform the severity of the patients’ pathologies; equally, other confounder variables such as type of feeding (enteral or parenteral) or the care bundle used to prevent VAP were not reported in all of the included studies, which could influence the results; in addition, is important to emphasize the prone position is usually used in ARDS patients admitted in ICU who may present higher APACHE-II scores and higher mortality rates than other ICU patients. Moreover, the main objective to be achieved with prone positioning in these patients with ARDS usually is to improve oxygenation and not so much to prevent VAPM. The second limitation is the use of various definitions to diagnosis VAP, as well as the reporting of VAP incidence using clinically suspected or microbiologically confirmed VAP or both, could have affected the results; third, the characteristics of the interventions ranged widely: supine position ranged from 0° to 25° of head-of-bed elevation, the duration in prone position ranged between 4 and 20 h daily, and the main outcomes were assessed at different end-points (7 days, 28 days, 90 days, etc.). Fourth, although heterogeneity was not important for the main outcomes, the various characteristics of the interventions and the different patients’ baseline pathologies could be a reason for the high clinical heterogeneity; five, the limited number of studies included and their sample size could influence the precision of the pooled estimate, especially for secondary outcomes; sixth, adverse events were not reported in most studies, and they could provide important information for deciding which body position to use; finally, the majority of the included studies were scored as “some concerns” in the risk of bias assessment, mainly due to lack of previous study protocol publications and nonreported deviations from the intended interventions (Additional files 14, 15).

## Conclusions

Semi-recumbent therapy seems to be the best position for reducing the VAP incidence, hospital length of stay and duration of MV in patients admitted to the ICU and undergoing MV. Prone is the most effective position to reduce the risk of mortality and the ICU length of stay in mechanically ventilated patients, but it has no effect on reducing the incidence of VAP. Regarding the optimal angle for preventing VAP when semi-recumbent, our study cautiously showed that higher degrees of head-of-bed elevation (60° angle or 30–60°) seemed to be better; however, this needs to be validated in additional rigorous trials.

## Supplementary Information

**Additional file 1.** Results of individual studies included.

**Additional file 2.** Risk ratio (95% CI) of the effect of different body positions on ventilator-associated pneumonia.

**Additional file 3.** Risk ratio (95% CI) of the effect of different body positions on mortality.

**Additional file 4.** Treatment ranking.

**Additional file 5.** Heterogeneity assessment.

**Additional file 6.** Funnel plots for asymmetry.

**Additional file 7.** Transitivity assessment.

**Additional file 8.** Inconsistency and incoherence assessment.

**Additional file 9.** Risk of bias assessment for included studies.

**Additional file 10.** Quality assessment—GRADE.

**Additional file 11.** Summary table results.

**Additional file 12.** Pooled effect sizes and 95% confidence interval (CI). Upper right triangle gives the pooled risk ratios for ventilator-associated pneumonia (column intervention relative to row), and lower left triangle pooled standardized mean differences from the network meta-analysis (row intervention relative to column).

**Additional file 13.** Treatment ranking for incidence of ventilator-associated pneumonia by different grades of semi-recumbent position.

**Additional file 14.** PRISMA CHECKLIST.

**Additional file 15.** Example of search strategy used in Pubmed database.

## Data Availability

Not applicable.
