# Supplementary material for: Body position for preventing ventilator-associated pneumonia for critically ill patients: a systematic review and network meta-analysis
Source: J Intensive Care. 2022 Feb 22;10:9. doi: 10.1186/s40560-022-00600-z (PMC8864849; doi:10.1186/s40560-022-00600-z)
Supplement: Supplementary file 1 — Additional file 1. Results of individual studies included. [file 40560_2022_600_MOESM1_ESM.docx]

| ADDITIONAL FILE 10. Quality assessment by GRADE. | | | | | | |  |  |
| --- | --- | --- | --- | --- | --- | --- | --- | --- |
| Certainty assessment | | | | | | | **Impact** | **Certainty** |
| № of studies | **Study design** | **Risk of bias** | **Inconsistency** | **Indirectness** | **Imprecision** | **Other considerations** |  |  |
| VAP | | | | | | | | |
| Prone | | | | | | | | |
| 6 | RCT | serious ^a^ | serious | serious ^d^ | very serious ^e^ | none | Meta-analysis: RR = 0.79 (95% CI: 0.57, 1.02)  NMA: SMD = -0.07 (95% CI: -0.27, 0.14) | VERY LOW |
| Semi-recumbent | | | | | | | | |
| 11 | RCT | very serious  ^b^ | not serious ^c^ | serious ^d^ | not serious | strong association | Meta-analysis: RR = 0.38 (95% CI: 0.25, 0.52)  NMA: SMD = -0.15 (95% CI: -0.30, 0.01) | LOW |
| Lateral Trendelenburg | | | | | | | | |
| 1 | RCT | serious ^a^ | serious | serious ^d^ | very serious ^e^ | none | Meta-analysis: RR = 3.86 (95% CI: -13.04, 20.76)  NMA: SMD = -0.18 (95% CI: -0.71, 0.35) | VERY LOW |
| MORTALITY | | | | | | | | |
| Prone | | | | | | | | |
| 4 | RCT | not serious | not serious ^c^ | serious ^d^ | very serious ^e^ | none | Meta-analysis: RR = 0.71 (95% CI: 0.50, 0.91)  NMA: SMD = -0.09 (95% CI: -0.20, 0.13) | VERY LOW |
| Semi-recumbent | | | | | | | | |
| 4 | RCT | very serious ^b^ | not serious ^c^ | not serious | very serious ^e^ | none | Meta-analysis: RR = 0.83 (95% CI: 0.53, 1.13)  NMA: SMD = -0.05 (95% CI: -0.13, 0.04) | VERY LOW |
| Lateral Trendelenburg | | | | | | | | |
| 1 | RCT | serious ^a^ | serious | serious ^d^ | very serious ^e^ | none | Meta-analysis: RR = 0.84 (95% CI: 0.64, 1.11)  NMA: SMD = 0.02 (95% CI: -0.15, 0.18) | VERY LOW |
| ICU LENGTH OF STAY | | | | | | | | |
| Prone | | | | | | | | |
| 4 | RCT | serious ^a^ | not serious ^c^ | serious ^d^ | very serious ^e^ | none | Meta-analysis: -0.89 days (95% CI: -6.49, 4.72)  NMA: SMD = -0.81 (95% CI: -7.72, 6.11) | VERY LOW |
| Semi-recumbent | | | | | | | | |
| 4 | RCT | very serious ^b^ | not serious ^c^ | not serious | serious ^f^ | none | Meta-analysis: 1.02 days (95% CI: -5.50, 7.54)  NMA: SMD = 1.09 (95% CI: -5.12, 7.29) | VERY LOW |
| Lateral Trendelenburg | | | | | | | | |
| 1 | RCT | serious ^a^ | serious | serious ^d^ | not serious | none | Meta-analysis: 1.25 days (95% CI: 0.90, 1.60)  NMA: SMD = -0.16 (95% CI: -13.40, 13.03) | VERY LOW |
| HOSPITAL LENGTH OF STAY | | | | | | | | |
| Prone | | | | | | | | |
| 1 | RCT | not serious | serious | serious ^d^ | very serious ^e^ | none | Meta-analysis: 5.8 days (95% CI: -8.25, 19.85)  NMA: SMD = 5.79 (95% CI: -24.41, 36.01) | VERY LOW |
| Semi-recumbent | | | | | | | | |
| 3 | RCT | very serious ^b^ | serious | serious ^d^ | serious ^f^ | none | Meta-analysis: -6.94 days (95% CI: -20.30, 6.43)  NMA: SMD = -7.29 (95% CI: -22.74, 8.17) | VERY LOW |
| Lateral Trendelenburg | | | | | | | | |
| 1 | RCT | serious ^a^ | serious | serious ^d^ | not serious | none | Meta-analysis: 1.25 days (95% CI: 0.58, 1.92)  NMA: SMD = -8.54 (95% CI: -39.14, 22.07) | VERY LOW |
| DURATION OF MECHANICAL VENTILATION | | | | | | | | |
| Prone | | | | | | | | |
| 3 | RCT | not serious | not serious ^c^ | serious ^d^ | very serious ^e^ | none | Meta-analysis: -2.83 days (95% CI: -8.03, 2.36)  NMA: SMD = -3.28 (95% CI: -8.05, 1.49) | VERY LOW |
| Semi-recumbent | | | | | | | | |
| 4 | RCT | very serious ^b^ | not serious ^c^ | not serious | serious ^f^ | none | Meta-analysis: 3.36 days (95% CI: -20.30, 6.43)  NMA: SMD = -3.26 (95% CI: -6.31, -0.20) | VERY LOW |
| Lateral Trendelenburg | | | | | | | | |
| 1 | RCT | serious ^a^ | serious | serious ^d^ | not serious | none | Meta-analysis: -0.50 days (95% CI: -0.73, -0.27)  NMA: SMD = -2.76 (95% CI: -9.43, 3.91) | VERY LOW |
| RCT: Randomized controlled trials; CI: Confidence interval; RR: Risk ratio; SMD: Standardized mean difference; NMA: Network meta-analysis.  a At least one study included had high risk of bias.  b Three o more studies included had high risk of bias.  c If at least 70% of studies included agree on the effect direction.  d If more than 50% of studies included had moderate or high indirectness.  e If more than 50% of studies included presented wide confidence intervals and not statistically significant effects.  f If more than 50% of studies included presented wide confidence intervals or not statistically significant effects  GRADE Working Group grades of evidence  High certainty: We are very confident that the true effect lies close to that of the estimate of the effect  Moderate certainty: We are moderately confident in the effect estimate: The true effect is likely to be close to the estimate of the effect, but there is a possibility that it is substantially different  Low certainty: Our confidence in the effect estimate is limited: The true effect may be substantially different from the estimate of the effect  Very low certainty: We have very little confidence in the effect estimate: The true effect is likely to be substantially different from the estimate of effect | | | | | | | | |
