# Supplementary material for: Body position for preventing ventilator-associated pneumonia for critically ill patients: a systematic review and network meta-analysis
Source: J Intensive Care. 2022 Feb 22;10:9. doi: 10.1186/s40560-022-00600-z (PMC8864849; doi:10.1186/s40560-022-00600-z)
Supplement: Supplementary file 2 — Additional file 2. Risk ratio (95% CI) of the effect of different body positions on ventilator-associated pneumonia. [file 40560_2022_600_MOESM2_ESM.docx]

| **ADDITIONAL FILE 11.** Summary findings table. | | | | | | | |
| --- | --- | --- | --- | --- | --- | --- | --- |
| **Body positions** | **Relative effect**  **(95% CI)** | **Anticipated absolute effect** | | **Nº of participants**  **(studies)** | **Relative effect ^Ψ^ (95% CI)**  **Network estimates** | **Certainty of evidence** | **Ranking (SUCRA)** |
|  |  | **Without intervention** | **With intervention** |  |  |  |  |
| **VAP** | | | | | | | |
| Semi-recumbent | RR 0.38  (0.24 to 0.52) | 308 per 1000 | 117 per 1000  (74 to 160) | 1326  (11 RCT) | -0.15 (-0.30 to 0.01) | ⨁⨁◯◯ Low | 1 |
| Prone | RR 0.79  (0.57 to 1.02) | 223 per 1000 | 183 per 1000  (123 to 241) | 751  (6 RCT) | -0.07 (-0.27 to 0.14) | ⨁◯◯◯  Very low | 3 |
| Supine | Reference comparator | No estimable | No estimable | No estimable | No estimable | Reference comparator | 4 |
| Lateral* | RR 0.13  (0.02 to 1.03) | 40 per 1000 | 5 per 1000  (1 to 41) | 395  (1 RCT) | -0.18 (-0.71 to 0.35)^¥^ | ⨁◯◯◯  Very low | 2 |
| **MORTALITY** | | | | | | | |
| Semi-recumbent | RR 0.83  (0.53 to 1.13) | 247 per 1000 | 205 per 1000  (131 to 280) | 575  (4 RCT) | -0.05 (-0.13 to 0.04) | ⨁◯◯◯  Very low | 2 |
| Prone | RR 0.71  (0.50 to 0.91) | 484 per 1000 | 339 per 1000  (242 to 440) | 267  (4 RCT) | -0.09 (-0.20 to 0.13) | ⨁◯◯◯  Very low | 1 |
| Supine | Reference comparator | No estimable | No estimable | No estimable | No estimable | Reference comparator | 3 |
| Lateral* | RR 1.27  (0.92 to 1.76) | 239 per 1000 | 303 per 1000  (220 to 420) | 395  (1 RCT) | 0.02 (-0.15 to 0.18)^¥^ | ⨁◯◯◯  Very low | 4 |
| **ICU LENGTH OF STAY** | | | | | | | |
| Semi-recumbent | - | - | MD 1.02 days  (-5.5 to 7.54) | 575  (4 RCT) | 1.09 (-5.12 to 7.29) | ⨁◯◯◯  Very low | 4 |
| Prone | - | - | MD -0.89 days  (-6.49 to 4.72) | 243  (4 RCT) | -0.81 (-7.72 to 6.11) | ⨁◯◯◯  Very low | 1 |
| Supine | Reference comparator | No estimable | No estimable | No estimable | No estimable | Reference comparator | 3 |
| Lateral* | - | - | MD -1.25 days  (-1.60 to -0.90) | 395  (1 RCT) | -0.16 (-13.40 to 13.03)^¥^ | ⨁◯◯◯  Very low | 2 |
| **HOSPITAL LENGTH OF STAY** | | | | | | | |
| Semi-recumbent | - | - | MD - 6.94 days  (-20.3 to 6.43) | 489  (3 RCT) | -7.29 (-22.74 to 8.17) | ⨁◯◯◯  Very low | 1 |
| Prone | - | - | MD 5.80 days  (-8.25 to 19.85) | 40  (1 RCT) | 5.79 (-24.41 to 36.01) | ⨁◯◯◯  Very low | 4 |
| Supine | Reference comparator | No estimable | No estimable | No estimable | No estimable | Reference comparator | 3 |
| Lateral* | - | - | MD -1.25 days  (-1.92 to -0.58) | 395  (1 RCT) | -8.54 (-39.14 to 22.07)^¥^ | ⨁◯◯◯  Very low | 2 |
| **DURATION OF MECHANICAL VENTILATION** | | | | | | | |
| Semi-recumbent | - | - | MD -3.36 days  (-4.27 to -0.37) | 458  (4 RCT) | -3.26 (-6.31 to -0.20) | ⨁◯◯◯  Very low | 1 |
| Prone | - | - | MD -2.83 days  (-8.03 to 2.36) | 131  (3 RCT) | -3.28 (-8.05 to 1.49) | ⨁◯◯◯  Very low | 2 |
| Supine | Reference comparator | No estimable | No estimable | No estimable | No estimable | Reference comparator | 4 |
| Lateral* | - | - | MD 0.50 days  (0.27 to 0.73) | 395  (1 RCT) | -2.76 (-9.43 to 3.91)^¥^ | ⨁◯◯◯  Very low | 3 |

*Comparison of lateral Trendelenburg (intervention group) versus semi-recumbent position. ¥ Network estimate of Lateral Trendelenburg versus supine position comparison. Ψ Relative effects expressed in standardized mean differences for VAP and mortality and in raw differences for ICU/hospital length of stay and duration of mechanical ventilation.

VAP, ventilator-associated pneumonia; ICU, intensive care unit; MD, mean difference; RCT, randomized controlled trial; CI, confidence interval; SUCRA, the surface under the cumulative ranking curve.
