# Supplementary material for: Body position for preventing ventilator-associated pneumonia for critically ill patients: a systematic review and network meta-analysis
Source: J Intensive Care. 2022 Feb 22;10:9. doi: 10.1186/s40560-022-00600-z (PMC8864849; doi:10.1186/s40560-022-00600-z)
Supplement: Supplementary file 3 — Additional file 3. Risk ratio (95% CI) of the effect of different body positions on mortality. [file 40560_2022_600_MOESM3_ESM.docx]

| **ADDITIONAL FILE 12.** Pooled effect sizes and 95% confidence interval (CI). Upper right triangle gives the pooled risk ratios for ventilator-associated pneumonia (column intervention relative to row), and lower left triangle pooled standardized mean differences from the network meta-analysis (row intervention relative to column). | | | | | | | | |
| --- | --- | --- | --- | --- | --- | --- | --- | --- |
| **Ventilator-associated pneumonia** | | |  | | |  | | |
|  | **Supine** | **Semi-recumbent**  **30** | | **Semi-recumbent**  **45** | **Semi-recumbent**  **30-45** | | **Semi-recumbent**  **60** | **Semi-recumbent**  **30-60** |
| **Supine** |  | **0.55 (0.22 to 0.88)** | | **0.39 (0.13 to 0.65)** | **0.35 (0.14 to 0.56)** | | NA | **0.23 (0.10 to 0.36)** |
| **Semi-recumbent**  **30** | -0.16 (-0.41 to 0.99) |  | | NA | NA | | NA | NA |
| **Semi-recumbent**  **45** | -0.17 (-0.43 to 0.08) | -0.02 (-0.33 to 0.31) | |  | NA | | 3.64 (-2.48 to 9.75) | NA |
| **Semi-recumbent**  **30-45** | 0.003 (-0.32 to 0.33) | 0.16 (-0.26 to 0.58) | | 0.17 (-0.24 to 0.59) |  | | NA | NA |
| **Semi-recumbent**  **60** | **-0.70 (-1.38 to -0.001)** | -0.54 (-1.26 to 0.17) | | -0.53 (-1.17 to 0.11) | -0.70 (-1.46 to 0.06) | |  | NA |
| **Semi-recumbent**  **30-60** | **-0.66 (-1.21 to -0.12)** | -0.50 (-1.10 to 0.97) | | -0.49 (-1.09 to 0.11) | **-0.66 (-1.29 to -0.03)** | | 0.04 (-0.84 to 0.92) |  |
| NA, not available. | | |  | | |  | | |
