# Supplementary material for: Body position for preventing ventilator-associated pneumonia for critically ill patients: a systematic review and network meta-analysis
Source: J Intensive Care. 2022 Feb 22;10:9. doi: 10.1186/s40560-022-00600-z (PMC8864849; doi:10.1186/s40560-022-00600-z)

**ADDITIONAL FILE 13**. Treatment ranking for incidence of ventilator-associated pneumonia by different grades of semi-recumbent position.


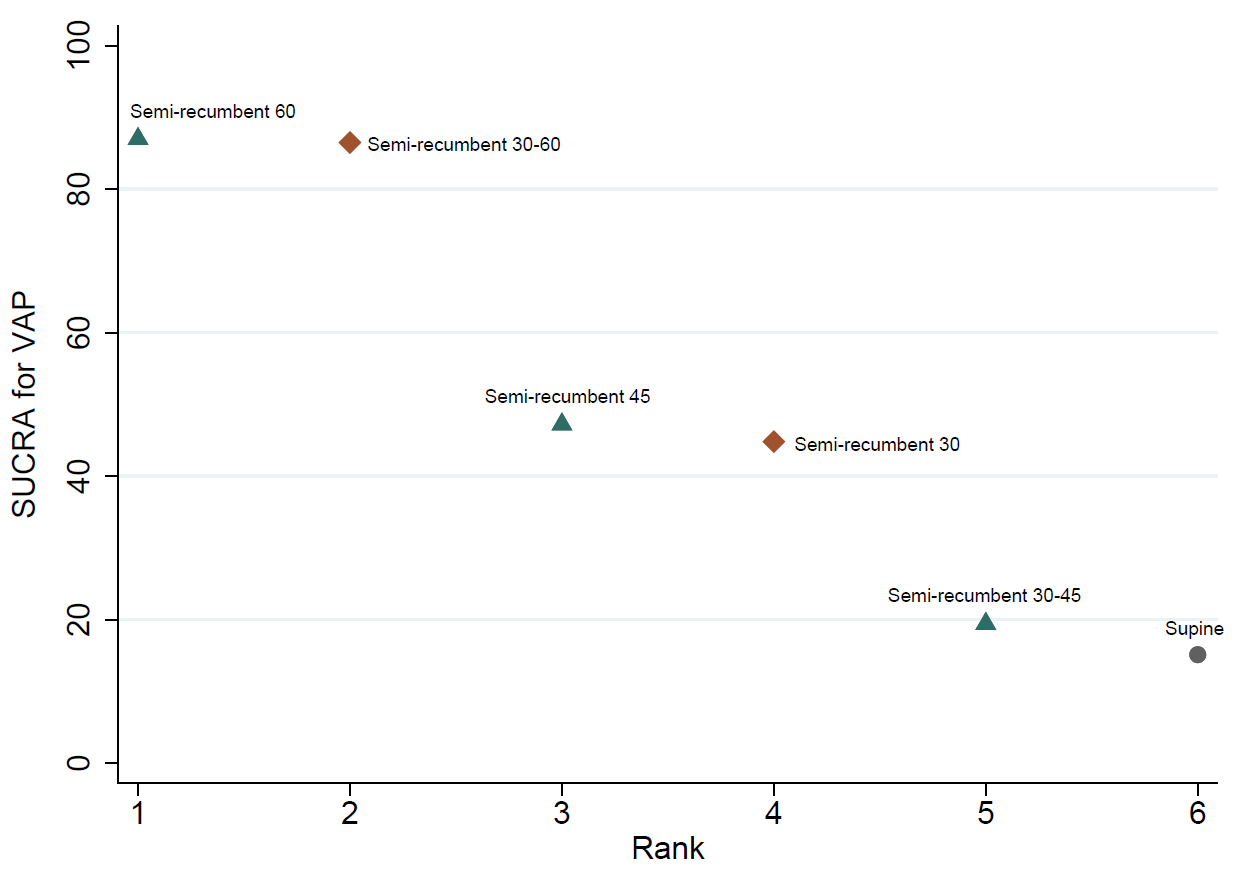

Supplement: Supplementary file 4 — Additional file 4. Treatment ranking. [file 40560_2022_600_MOESM4_ESM.docx]
