# Supplementary material for: Body position for preventing ventilator-associated pneumonia for critically ill patients: a systematic review and network meta-analysis
Source: J Intensive Care. 2022 Feb 22;10:9. doi: 10.1186/s40560-022-00600-z (PMC8864849; doi:10.1186/s40560-022-00600-z)
Supplement: Supplementary file 6 — Additional file 6. Funnel plots for asymmetry. [file 40560_2022_600_MOESM6_ESM.docx]

**ADDITIONAL FILE 15. Example of search strategy used in Pubmed database.**

"posture"[MeSH Terms] OR "posture"[All Fields] OR ("body"[All Fields] AND "position"[All Fields]) OR "body position"[All Fields] OR ("patient positioning"[MeSH Terms] OR ("patient"[All Fields] AND "positioning"[All Fields]) OR "patient positioning"[All Fields] OR "positioning"[All Fields] OR "position"[All Fields] OR "positions"[All Fields] OR "positional"[All Fields] OR "positioned"[All Fields] OR "positionings"[All Fields] OR "positions"[All Fields]) OR "prone position"[MeSH Terms] OR "head down tilt"[MeSH Terms] OR "supine position"[MeSH Terms] OR "semi-recumbent"[All Fields] OR ("semirecumbency"[All Fields] OR "semirecumbent"[All Fields])) AND ("prevent"[All Fields] OR "preventability"[All Fields] OR "preventable"[All Fields] OR "preventative"[All Fields] OR "preventatively"[All Fields] OR "preventatives"[All Fields] OR "prevented"[All Fields] OR "preventing"[All Fields] OR "prevention and control"[MeSH Subheading] OR ("prevention"[All Fields] AND "control"[All Fields]) OR "prevention and control"[All Fields] OR "prevention"[All Fields] OR "prevention s"[All Fields] OR "preventions"[All Fields] OR "preventive"[All Fields] OR "preventively"[All Fields] OR "preventives"[All Fields] OR "prevents"[All Fields] OR "prevent*"[All Fields]) AND ("pneumonia, ventilator associated"[MeSH Terms] OR ("pneumonia"[All Fields] AND "ventilator associated"[All Fields]) OR "ventilator-associated pneumonia"[All Fields] OR ("ventilator"[All Fields] AND "associated"[All Fields] AND "pneumonia"[All Fields]) OR "ventilator associated pneumonia"[All Fields])
