# Supplementary material for: Body position for preventing ventilator-associated pneumonia for critically ill patients: a systematic review and network meta-analysis
Source: J Intensive Care. 2022 Feb 22;10:9. doi: 10.1186/s40560-022-00600-z (PMC8864849; doi:10.1186/s40560-022-00600-z)
Supplement: Supplementary file 7 — Additional file 7. Transitivity assessment. [file 40560_2022_600_MOESM7_ESM.docx]

| **ADDITIONAL FILE 1.** Results of individual studies included. | | | | | |
| --- | --- | --- | --- | --- | --- |
| **Study, year** | **Incidence of VAP** | **Mortality** | **ICU length of stay** | **Hospital length of stay** | **Duration of MV** |
| *Ayzac et al, 2016*  ARM 1: Supine  ARM 2: Prone | ARM 1: 41 (44.1%)  ARM 2: 52 (55.9%) |  |  |  |  |
| *Bassi et al, 2017*  ARM 1: Lateral Trendelenburg  ARM 2: Semi-recumbent 30º | ARM 1: 1 (0.5%)  ARM 2: 8 (4%) | ARM 1: 59 (30.4%)  ARM 2: 48 (23.9%) | ARM 1: 7.75 ± 1.5  ARM 2: 9 ± 2 | ARM 1: 16.5 ± 3.33  ARM 2: 17.75 ± 3.5 | ARM 1: 5.25 ± 1.17  ARM 2: 4.75 ± 1.17 |
| *Beuret et al, 2002*  ARM 1: Supine 20º  ARM 2: Prone | ARM 1: 11 (38.4%)  ARM 2: 5 (20%) | ARM 1: 12 (46%)  ARM 2: 7 (28%) | ARM 1: 19.4 ± 24.1  ARM 2: 16.5 ± 12.9 |  | ARM 1: 14.6 ± 17.7  ARM 2: 12.7 ± 10 |
| *Cai et al, 2006*  ARM 1: Supine 0º  ARM 2: Semi-recumbent 30º | ARM 1: 13 (48.1%)  ARM 2: 4 (14.8%) |  |  |  |  |
| *Drakulovic et al, 1999*  ARM 1: Supine 0º  ARM 2: Semi-recumbent | ARM 1: 11 (23%)  ARM 2: 2 (5%) | ARM 1: 13 (27.7%)  ARM 2: 7 (17.9%) | ARM 1: 9.7 ± 7.8  ARM 2: 9.3 ± 7.2 |  | ARM 1: 7.1 ± 7  ARM 2: 6 ± 6.2 |
| *Fernández et al, 2008*  ARM 1: Supine  ARM 2: Prone | ARM 1: 1 (5%)  ARM 2: 3 (14%) | ARM 1: 10 (52.6%)  ARM 2: 8 (38.1%) | ARM 1: 17.5 ± 16.1  ARM 2: 14.7 ± 9.7 | ARM 1: 25.5 ± 17.4  ARM 2: 31.3 ± 26.4 | ARM 1: 15.7 ± 16.9  ARM 2: 11.9 ± 9.2 |
| *Guérin et al, 2004*  ARM 1: Semi-recumbent 30º  ARM 2: Prone | ARM 1: 91 (24.1%)  ARM 2: 85 (20.6%) | ARM 1: 119 (31.5%)  ARM 2: 134 (32.4%) |  |  | ARM 1: 14.1 ± 8.6  ARM 2: 13.7 ± 7.8 |
| *Hadi Hassankhan et al, 2017*  ARM 1: Semi-recumbent 45º  ARM 2: Semi-recumbent 60º | ARM 1: 8 (72.7%)  ARM 2: 2 (20%) |  |  |  |  |
| *Hang et al, 2012*  ARM 1: Supine  ARM 2: Semi-recumbent 30º-45º | ARM 1: 9 (47.3%)  ARM 2: 3 (15%) | ARM 1: 9 (47.3%)  ARM 2: 5 (25%) | ARM 1: 18 ± 12.1  ARM 2: 11.3 ± 7.9 | ARM 1: 71.1 ± 10.6  ARM 2: 48.8 ± 9 | ARM 1: 10.1 ± 7.1  ARM 2: 6 ± 4.5 |
| *Hu et al, 2012*  ARM 1: Supine  ARM 2: Semi-recumbent 30º-45º | ARM 1: 21 (48.8%)  ARM 2: 8 (18.6%) |  |  |  |  |
| *Keely et al, 2007*  ARM 1: Supine  ARM 2: Semi-recumbent 45º | ARM 1: 5 (38%)  ARM 2: 4 (24%) |  |  |  |  |
| *Loan et al, 2012*  ARM 1: Supine  ARM 2: Semi-recumbent 30º | ARM 1: 19 (17.0%)  ARM 2: 26 (22.2%) | ARM 1: 11 (9.8%)  ARM 2: 17 (14.5%) | ARM 1: 24.5 ± 10.7  ARM 2: 35.8 ± 17.83 | ARM 1: 44.5 ± 19.7  ARM 2: 42.3 ± 17.5 |  |
| *Mancebo et al, 2006*  ARM 1: Supine  ARM 2: Prone | ARM 1: 9 (15%)  ARM 2: 14 (18.4%) | ARM 1: 35 (58%)  ARM 2: 33 (43%) | ARM 1: 22 ± 14.1  ARM 2: 27.9 ± 18.5 |  |  |
| *Tahereh Najafi Ghezeljeh et al, 2017*  ARM 1: Supine  ARM 2: Semi-recumbent 30º  ARM 3: Semi-recumbent 45º | ARM 1: 21 (52.5%)  ARM 2: 13 (32.5%)  ARM 3: 8 (20%) |  |  |  |  |
| *Van Nieuwenhoven et al, 2006*  ARM 1: Supine  ARM 2: Semi-recumbent 45º | ARM 1: 8 (7%)  ARM 2: 13 (12%) | ARM 1: 38 (34.9%)  ARM 2: 39 (34.8%) | ARM 1: 10 ± 12  ARM 2: 9 ± 12 | ARM 1: 24 ± 11  ARM 2: 27 ± 11 | ARM 1: 6 ± 7  ARM 2: 6 ± 7 |
| *Voggenreiter et al, 2005*  ARM 1: Supine  ARM 2: Prone | ARM 1: 17 (89%)  ARM 2: 13 (62%) | ARM 1: 3 (16%)  ARM 2: 1 (5%) |  |  | ARM 1: 33 ± 23  ARM 2: 30 ± 17 |
| *Watanabe et al, 2002*  ARM 1: Supine  ARM 2: Prone | ARM 1: 2 (25%)  ARM 2: 1 (12.5%) |  | ARM 1: 17.2 ± 3.4  ARM 2: 12.8 ± 4.4 |  |  |
| *Wu et al, 2009*  ARM 1: Supine  ARM 2: Semi-recumbent 30º-60º | ARM 1: 48 (85.7%)  ARM 2: 11 (19.6%) |  |  |  | ARM 1: 12.8 ± 5.8  ARM 2: 4.6 ± 3.8 |
| *Xue et al, 2012*  ARM 1: Supine  ARM 2: Semi-recumbent 30º-45º | ARM 1: 12 (25%)  ARM 2: 4 (8.3%) |  |  |  |  |
| *Yu et al, 2012*  ARM 1: Supine  ARM 2: Semi-recumbent 30º | ARM 1: 14 (43.7%)  ARM 2: 5 (15.1%) |  |  |  |  |
