# Supplementary material for: Body position for preventing ventilator-associated pneumonia for critically ill patients: a systematic review and network meta-analysis
Source: J Intensive Care. 2022 Feb 22;10:9. doi: 10.1186/s40560-022-00600-z (PMC8864849; doi:10.1186/s40560-022-00600-z)

**ADDITIONAL FILE 2.** Risk ratio (95% CI) of the effect of different body positions on ventilator-associated pneumonia.


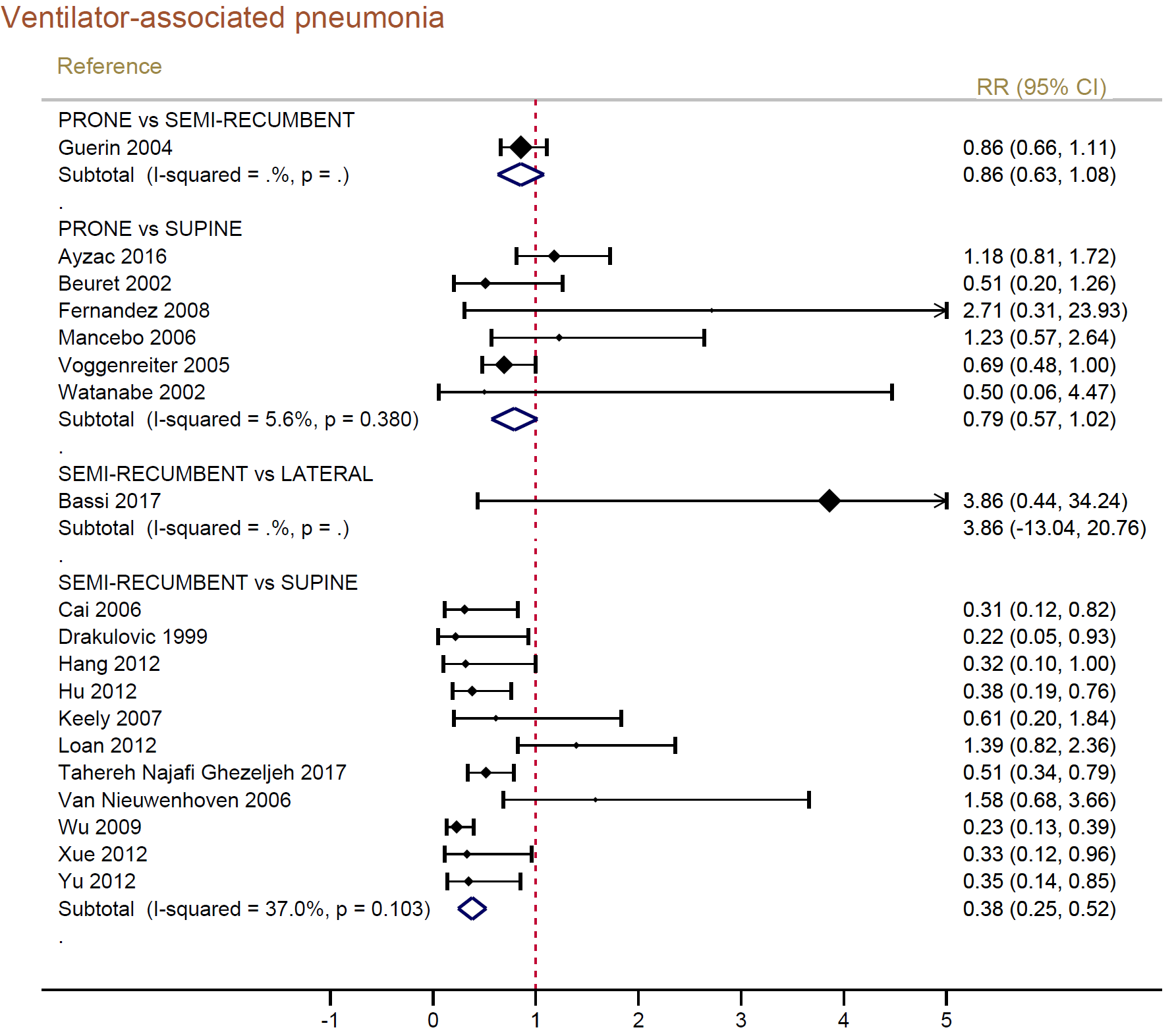

Supplement: Supplementary file 8 — Additional file 8. Inconsistency and incoherence assessment. [file 40560_2022_600_MOESM8_ESM.docx]
