# Supplementary material for: Body position for preventing ventilator-associated pneumonia for critically ill patients: a systematic review and network meta-analysis
Source: J Intensive Care. 2022 Feb 22;10:9. doi: 10.1186/s40560-022-00600-z (PMC8864849; doi:10.1186/s40560-022-00600-z)
Supplement: Supplementary file 10 — Additional file 10. Quality assessment—GRADE. [file 40560_2022_600_MOESM10_ESM.docx]

**ADDITIONAL FILE 4.** Treatment ranking table.

|  | | | | | | | | | | |
| --- | --- | --- | --- | --- | --- | --- | --- | --- | --- | --- |
| ***Group*** | **Ventilator-associated pneumonia** | | **Mortality** | | **ICU length of stay** | | **Hospital length of stay** | | **Duration of mechanical ventilation** | |
|  | **SUCRA** | **Rank** | **SUCRA** | **Rank** | **SUCRA** | **Rank** | **SUCRA** | **Rank** | **SUCRA** | **Rank** |
| **SUPINE** | 18.6 | 0.6 | 26.1 | 1.8 | 51.2 | 15.6 | 37.1 | 6.5 | 10.6 | 0.3 |
| **SEMI-RECUMBENT** | 71.4 | 32.1 | 61.1 | 13.5 | 37.6 | 9.6 | 68.9 | 31.5 | 67.6 | 27.0 |
| **PRONE** | 44.8 | 14.8 | 89.3 | 76.5 | 59.3 | 36.7 | 28.2 | 14.8 | 65.7 | 38.6 |
| **LATERAL** | 65.3 | 52.4 | 23.5 | 8.2 | 51.9 | 38.2 | 65.8 | 47.1 | 56.2 | 34.1 |
| ICU, intensive care unit; SUCRA, the surface under the cumulative ranking curve. | | | | | | | | | | |
