# Supplementary material for: Body position for preventing ventilator-associated pneumonia for critically ill patients: a systematic review and network meta-analysis
Source: J Intensive Care. 2022 Feb 22;10:9. doi: 10.1186/s40560-022-00600-z (PMC8864849; doi:10.1186/s40560-022-00600-z)
Supplement: Supplementary file 11 — Additional file 11. Summary table results. [file 40560_2022_600_MOESM11_ESM.docx]

**ADDITIONAL FILE 5.** Heterogeneity assessment table.

|  |  |  | | | | | |  |
| --- | --- | --- | --- | --- | --- | --- | --- | --- |
| **Outcome** | **Semi-recumbent**  **Vs**  **Supine** | | | | **Prone**  **Vs**  **Supine** | | | |
|  | **I^2^** | | **p** | **Tau^2^** | **I^2^** | **p** | **Tau^2^** | |
| **VAP** | 37% | | 0.103 | 0.02 | 5.6% | 0.380 | 0.01 | |
| **Mortality** | 15.2% | | 0.316 | 0.02 | 0.0% | 0.895 | 0.00 | |
| **ICU length of stay** | 91% | | <0.0001 | 39.51 | 66.4% | 0.030 | 20.51 | |
| **Hospital length of stay** | 96.2% | | <0.0001 | 133.45 | - | - | - | |
| **Duration of MV** | 92.9% | | <0.0001 | 18.81 | 0.0% | 0.948 | 0.00 | |

ICU, intensive care unit; MV, mechanical ventilation.
