# Supplementary material for: Body position for preventing ventilator-associated pneumonia for critically ill patients: a systematic review and network meta-analysis
Source: J Intensive Care. 2022 Feb 22;10:9. doi: 10.1186/s40560-022-00600-z (PMC8864849; doi:10.1186/s40560-022-00600-z)
Supplement: Supplementary file 12 — Additional file 12. Pooled effect sizes and 95% confidence interval (CI). Upper right triangle gives the pooled risk ratios for ventilator-associated pneumonia (column intervention relative to row), and lower left triangle pooled standardized mean differences from the network meta-analysis (row intervention relative to column). [file 40560_2022_600_MOESM12_ESM.docx]

**ADDITIONAL FILE 6.** Funnel plots for asymmetry.


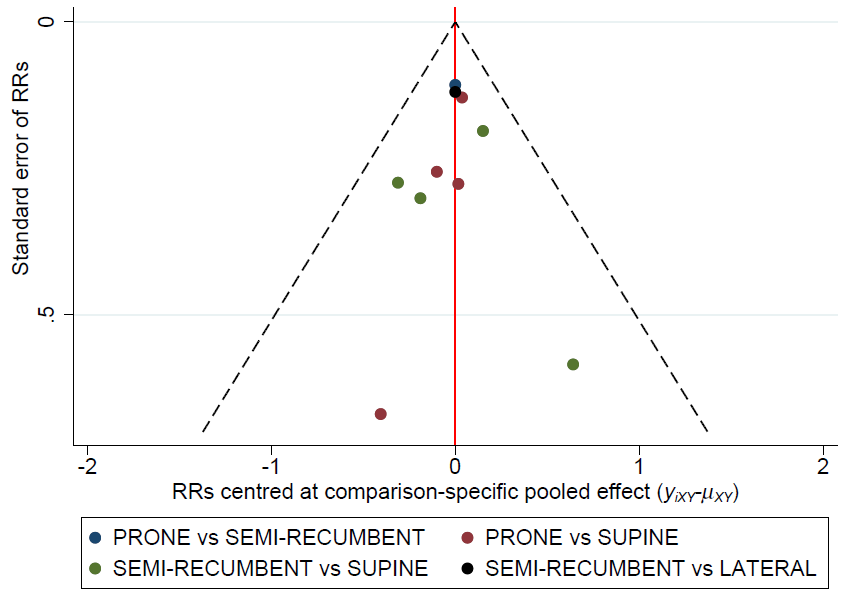


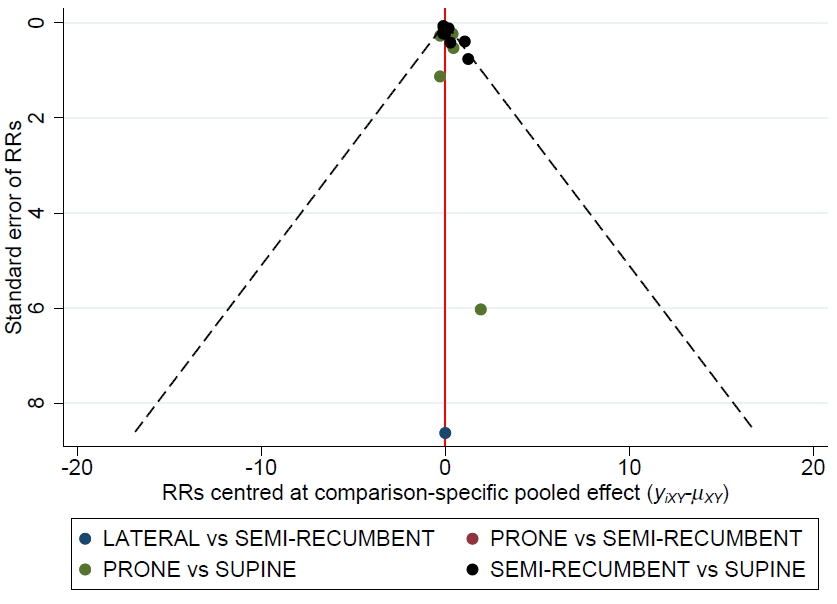


**MORTALITY**

**VAP**


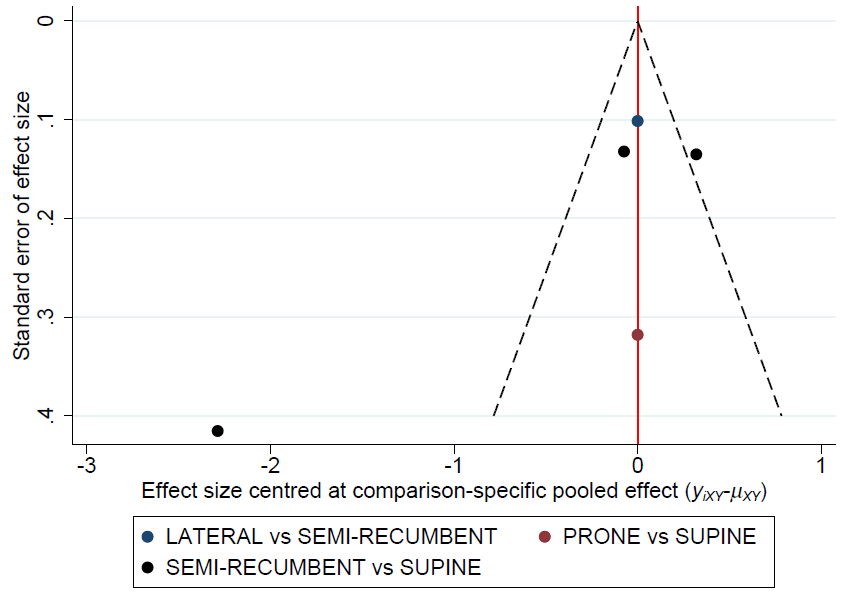

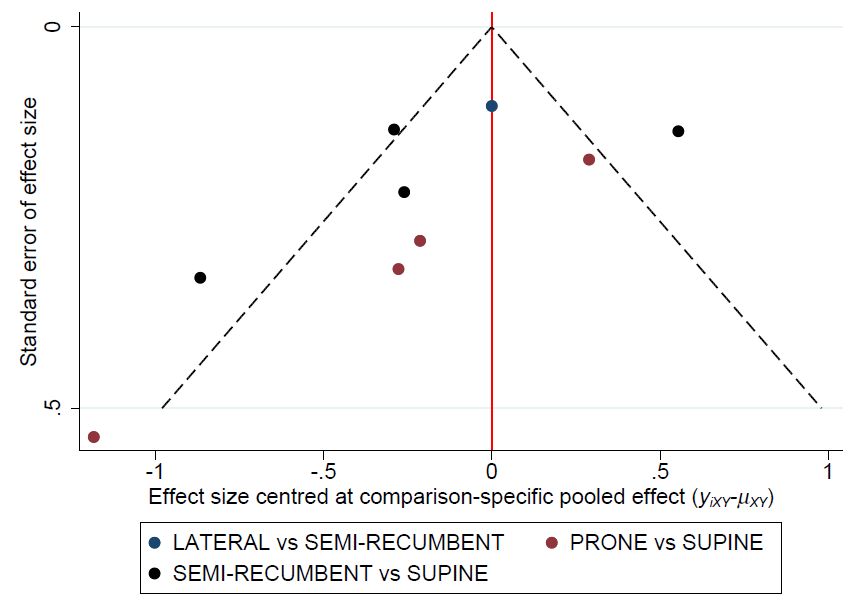


**HOSPITAL LENGTH OF STAY**

**ICU LENGTH OF STAY**


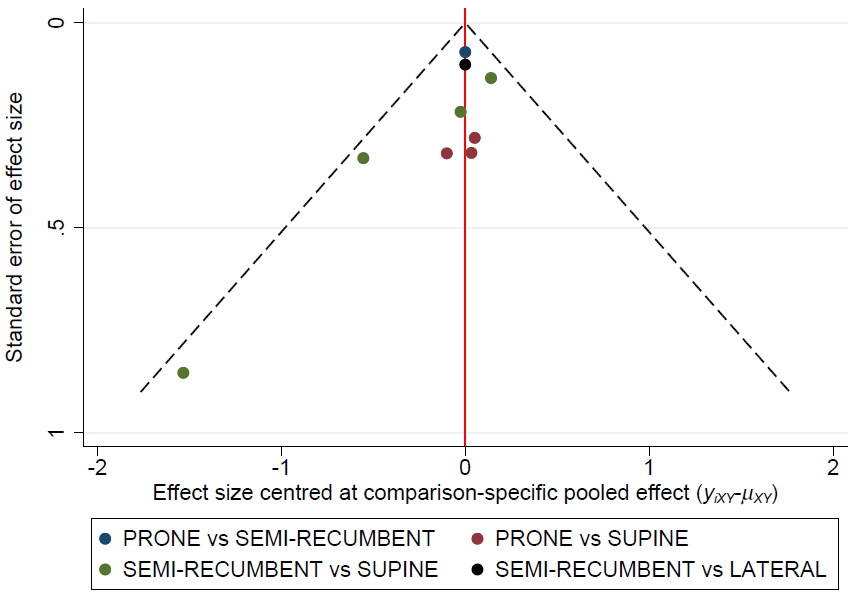


**DURATION OF MECHANICAL VENTILATION**
