# Supplementary material for: Body position for preventing ventilator-associated pneumonia for critically ill patients: a systematic review and network meta-analysis
Source: J Intensive Care. 2022 Feb 22;10:9. doi: 10.1186/s40560-022-00600-z (PMC8864849; doi:10.1186/s40560-022-00600-z)
Supplement: Supplementary file 13 — Additional file 13. Treatment ranking for incidence of ventilator-associated pneumonia by different grades of semi-recumbent position. [file 40560_2022_600_MOESM13_ESM.docx]

**ADDITIONAL FILE 7.** Transitivity assessment table.

|  | | | |
| --- | --- | --- | --- |
|  | **Supine vs Prone position** | **Supine vs Semi-recumbent** | **p- value** |
| **Events** | 8 ± 6.63 | 12.8 ± 6.94 | 0.296 |
| **N total** | 26.8 ± 19.87 | 63 ± 42.57 | 0.139 |
| **Female** | 9.6 ± 6.65 | 21 ± 13.48 | 0.140 |
| **Baseline Age** | 54.06 ± 7.15 | 59.20 ± 14.24 | 0.498 |
| **Baseline APACHE II** | - | 22.86 ± 4.6 | - |
| **Baseline SAPS II** | 42.23 ± 14.66 | - | - |
| **Baseline PaO^2^/FiO^2^** | 212.08 ± 73.48 | - | - |
| **Baseline GCS score** | 5.6 ± 2.2 | 9.4 ± 4.3 | - ***** |
| APACHE, Acute Physiology and Chronic Health disease Classification System; SAPS, Simplified Acute Physiology Score; GCS, Glasgow Coma Scale.  *Mean difference was not calculated because only one study in each group (prone and semi-recumbent) reported the GCS score. | | | |
