# Supplementary material for: Body position for preventing ventilator-associated pneumonia for critically ill patients: a systematic review and network meta-analysis
Source: J Intensive Care. 2022 Feb 22;10:9. doi: 10.1186/s40560-022-00600-z (PMC8864849; doi:10.1186/s40560-022-00600-z)
Supplement: Supplementary file 14 — Additional file 14. PRISMA CHECKLIST. [file 40560_2022_600_MOESM14_ESM.docx]

**ADDITIONAL FILE 8. Inconsistency and incoherence assessment.**

**Incoherence report for VAP.** SR: semi-recumbent; NA: not applicable. Global test based on a random-effects design by treatment interaction model. X^2^ statistic: 3.862 (1 degrees of freedom); p value: 0.049. Relative effect estimates below -0.200 and above 0.200 are considered clinically important.

| Comparison | Evidence | NMA SMD | Direct SMD | Indirect SMD | Difference of SMD (inconsistency) | P value | Incoherence judgment |
| --- | --- | --- | --- | --- | --- | --- | --- |
| Lateral-SR | Direct | **-** | -3.86 (-20.76 to 13.04) | **-** | NA | - | Major concerns |
| Prone-SR | Mixed | **0.60** (0.36 to 0.84) | **0.86** (0.48 to 1.23) | **0.43** (0.11 to 0.74) | 0.43 (-0.06 to 0.92) | 0.081 | Some concerns |
| Prone-Supine | Mixed | **0.95** (0.72 to 1.18) | **0.82** (0.54 to 1.09) | **1.24** (0.84 to 1.65) | -0.43 (-0.92 to 0.06) | 0.081 | No concerns |
| SR-Supine | Mixed | **0.35** (0.21 to 0.49) | **0.39** (0.24 to 0.54) | -0.05 (-0.51 to 0.42) | 0.43 (-0.06 to 0.92) | 0.081 | Major concerns |
| Lateral-Prone | Indirect | - | - | -4.46 (-21.36 to 12.44) | NA | - | Major concerns |
| Lateral-Supine | Indirect | - | - | -3.51 (-20.41 to 13.39) | NA |  | Major concerns |

**Incoherence report for Mortality.** SR: semi-recumbent; NA: not applicable. Global test based on a random-effects design by treatment interaction model. X^2^ statistic: 33.379 (1 degrees of freedom); p value: 0.000. Relative effect estimates below -0.200 and above 0.200 are considered clinically important.

| Comparison | Evidence | NMA SMD | Direct SMD | Indirect SMD | Difference of SMD (inconsistency) | P value | Incoherence judgment |
| --- | --- | --- | --- | --- | --- | --- | --- |
| Lateral-SR | Direct | **-** | **-0.84** (-1.08 to -0.61) | **-** | NA | - | Major concerns |
| Prone-SR | Mixed | **0.70** (0.52 to 0.88) | **1.03** (0.82 to 1.24) | -0.13 (-0.47 to 0.20) | **1.16** (0.77 to 1.56) | **0.000** | Major concerns |
| Prone-Supine | Mixed | **1.02** (0.85 to 1.20) | **0.71** (0.50 to 0.91) | 1.87 (1.53 to 2.21) | **-1.16** (-1.56 to -0.77) | **0.000** | No concerns |
| SR-Supine | Mixed | **0.33** (0.13 to 0.52) | **0.84** (0.58 to 1.10) | **-0.32** (-0.62 to -0.03) | **1.16** (0.77 to 1.56) | **0.000** | Major concerns |
| Lateral-Prone | Indirect | - | - | -1.54 (-1.84 to -1.25) | NA | - | Major concerns |
| Lateral-Supine | Indirect | - | - | -0.52 (-0.82 to -0.21) | NA |  | Major concerns |

**Incoherence report for UCI length of stay.** SR: semi-recumbent; NA: not applicable. Global test based on a random-effects design by treatment interaction model. X^2^ statistic: 0.00 (0 degrees of freedom); p value: NA. Relative effect estimates below -1.00 and above 1.00 (raw mean difference) are considered clinically important.

| Comparison | Evidence | NMA SMD | Direct SMD | Indirect SMD | Difference of SMD (inconsistency) | P value | Incoherence judgment |
| --- | --- | --- | --- | --- | --- | --- | --- |
| Lateral-SR | Direct | **-** | -1.25 (-12.75 to 10.25) | **-** | NA | - | Major concerns |
| Prone-Supine | Direct | - | -0.91 (-7.64 to 5.83) | - | NA | - | Major concerns |
| SR-Supine | Direct | - | 1.05 (-5.08 to 7.17) | - | NA | - | Major concerns |
| Lateral-Prone | Indirect | - | **-** | 0.70 (-13.97 to -15.37) | NA | - | Major concerns |
| Lateral-Supine | Indirect | - | - | -0.21 (-13.23 to 12.82) | NA | - | Major concerns |
| Prone-SR | Indirect | - | - | -1.95 (-11.06 to 7.15) | NA | - | Major concerns |

**Incoherence report for Hospital length of stay.** SR: semi-recumbent; NA: not applicable. Global test based on a random-effects design by treatment interaction model. X^2^ statistic: 0.00 (0 degrees of freedom); p value: NA. Relative effect estimates below -2.00 and above 2.00 are considered clinically important.

| Comparison | Evidence | NMA SMD | Direct SMD | Indirect SMD | Difference of SMD (inconsistency) | P value | Incoherence judgment |
| --- | --- | --- | --- | --- | --- | --- | --- |
| Lateral-SR | Direct | **-** | -1.25 (-23.87 to 21.37) | **-** | NA | - | Major concerns |
| Prone-Supine | Direct | - | 5.80 (-20.81 to 32.41) | - | NA | - | Major concerns |
| SR-Supine | Direct | - | -6.94 (-20.28 to 6.41) | - | NA | - | Major concerns |
| Lateral-Prone | Indirect | - | **-** | 13.99 (-51.38 to 23.41) | NA | - | Major concerns |
| Lateral-Supine | Indirect | - | - | -8.19 (-34.45 to 18.08) | NA | - | Major concerns |
| Prone-SR | Indirect | - | - | 12.74 (-17.04 to 42.51) | NA | - | Major concerns |

**Incoherence report for MV length.** SR: semi-recumbent; NA: not applicable. Global test based on a random-effects design by treatment interaction model. X^2^ statistic: 0.023 (1 degrees of freedom); p value: 0.88. Relative effect estimates below -1.00 and above 1.00 are considered clinically important.

| Comparison | Evidence | NMA SMD | Direct SMD | Indirect SMD | Difference of SMD (inconsistency) | P value | Incoherence judgment |
| --- | --- | --- | --- | --- | --- | --- | --- |
| Lateral-SR | Direct | - | 0.50 (-7.15 to 8.15) | - | NA | - | No concerns |
| Prone-SR | Mixed | 0.04 (-5.53 to 5.60) | -0.40 (-8.13 to 7.33) | 0.50 (-7.52 to 8.52) | -0.90 (12.04 to 10.24) | 0.874 | No concerns |
| Prone-Supine | Mixed | -3.21 (8.64 to 2.21) | -2.86 (-9.79 to 4.06) | -3.76 (12.49 to 4.96) | 0.90 (-10.24 to 12.04) | 0.874 | No concerns |
| SR-Supine | Mixed | -3.25 (-7.01 to 0.52) | -3.36 (-7.40 to 0.68) | -2.46 (-12.84 to 7.92) | -0.90 (12.04 to 10.24) | 0.874 | No concerns |
| Lateral-Prone | Indirect | - | - | 0.47 (-8.99 to 9.93) | NA | - | No concerns |
| Lateral-Supine | Indirect | - | - | -2.75 (-11.27 to 5.78) | NA |  | No concerns |
