# Supplementary material for: Body position for preventing ventilator-associated pneumonia for critically ill patients: a systematic review and network meta-analysis
Source: J Intensive Care. 2022 Feb 22;10:9. doi: 10.1186/s40560-022-00600-z (PMC8864849; doi:10.1186/s40560-022-00600-z)
Supplement: Supplementary file 15 — Additional file 15. Example of search strategy used in Pubmed database. [file 40560_2022_600_MOESM15_ESM.docx]

**ADDITIONAL FILE 9. Risk of bias assessment for included studies.**
